# Supplementary material for: Animals as Reservoir for Human Norovirus
Source: Viruses. 2019 May 25;11(5):478. doi: 10.3390/v11050478 (PMC6563253; doi:10.3390/v11050478)
Supplement: Supplementary file 1 [file viruses-11-00478-s001.pdf]

Supplementary information

**Supplementary Table 1** Studies investigating animal norovirus in animals and the detection methods that were applied. Included are studies that analyzed faecal samples by RT-PCR, real-time RT-PCR or EM and serological studies. Frequently used primers sets are p289/p290 and p290/p110 (universal calicivirus primers, RdRp), JV12/JV13 (norovirus specific), PNV7/PNV8 (porcine norovirus specific, RdRp), G2SKF/G2SKR (GII, capsid), MON432/MON477 (norovirus), FNoV-F9 and FNoVR15 (GIV specific), JV102/JV103 (canine norovirus specific), CBeCu-F/R (bovine norovirus specific). Unless otherwise stated sample were collected at one time point. a=longitudinal study, b=case control, c=outbreak. A=adult (in pigs >6month), J=juvenile

| Location | Host | Investigated genogroup, genotye | Animal norovirus virus detected | Prevalence (%) | Detection method | Primers, probes and antigens used | Sample collection: Sample type Faeces (F) Pooled Faeces (PF) Sera (S) Intestinal content (IC) | Sample collection: Location (n) | Age Clinical status Adult (A) Juvenile (J) Symptomatic (+) Asymptomatic (-) | Ref |
|---|---|---|---|---|---|---|---|---|---|---|
| **The Americas** | | | | | | | | | | |
| USA | Pigs | GII.11/18/19 | GII.11/18/19 | 64/621 (10)[1] | RT-PCR, microwell hybridization | PNV7/PNV8, Hybridization probes specific for GII.11/18/19 | F[a] | Farms #7 Abattoirs #1 | J/A- | [191] |
| | Pigs | GII.11 | GII.11 | 78/110 (71) | ELISA | VLPs of GII.11 (SW918) | S | Farms Abattoirs #1 | NA | [46] |
| | | calicivirus | none | 0/104 (0) | RT-PCR | p289H,I-p290H,I,J,K | F/IC | Farms #1 Abattoirs #2 | J- | |
| | Pigs | GII.11/18/19 | GII.11/18 | 78/413 (19) | RT-PCR, microwell hybridization | PNV7/PNV8, G2SKF/G2SKR. Hybridization probes specific for GII.11/GII.18/GII.19 | PF | Farms #9 | A- | [28] |
| | Pigs | calicivirus | GII.11/18/19 | 6/275 (2) | RT-PCR | p290/p110 | F | Farms #6 Abattoirs #1 | A- | [25] |
| | Pigs | calicivirus | none | 0/139 (0) | RT-PCR | p290/p110, G2SKFM-Po/G2SKRM-Po, JV12Y/JV13I | F | Farms #3 | J | [48] |

| | | | | | | | | | | |
|---|---|---|---|---|---|---|---|---|---|---|
| | Cattle | GIII.2 | GIII.2 | 231/231 (100) | ELISA | VLPs of GIII.2 (AF542084) | S[a] | Feedlots #3 | J | [49]* |
| | Cattle | calicivirus | GIII.1/2 | 54/75 (72) | RT-PCR | NV35/36, p290/p289 JV12/13 J11 U/L, J21 U/L, BEC-POL5/3, NBU-F/R, CBECU-F/R, | RS[a,b] | Farms #2 | J | [52]* |
| | Cattle | GIII | GIII | 128/444 (29) | real-time RT-PCR | BNoV-fwd/rev (1014) | F[b] | Farms #140 | J+/- | [50] |
| | Cattle | norovirus | GIII.1/2 | 52/74 (70) | RT-PCR | MON432/MON477, CDC primer set | F | Farms #22 | J+ | [51] |
| | cats | calicivirus | GIV.2 | 6/24 (43) | RT-PCR | p289/p290, FNoV-F9/FNoVR15 | F[c] | Clinic #1 Shelter #1 | J/A+/- | [53] |
| | cats | all | GIV | 1/6 (17) | RT-PCR | metagenomics, random primers | PF | Shelter #1 | NA | [54] |
| | Sea lion | all | GII/GIV | 4/47 (9) | RT-PCR | metagenomics, random primers | F | Marine mammal centre #1 | J/A | [55] |
| Canada | Pigs | norovirus | GII.11/18 | 26/120 (22) | RT-PCR | Region B and A primer MON431-MON434 (GI, GII), G1/G2 (SRSV) | PF[a] | Farms #10 | NA | [30] |
| | Cattle | norovirus | GIII.2 | 2/179 (1) | | | PF | Farms #45 | J/A | |
| | Pigs | calicivirus | GII.11/18 | 17/20 (85) [2] | RT-PCR | p289/p290, p289N/p290N | PF/F | Farms #20 | J/A | [31] |
| | Pigs | | | 12/66 (18) | | | IC[3] | Abattoirs #2 | A- | |
| | Pigs | calicivirus | porcine NoV | 4/200 (2) | real-time RT-PCR, RT-PCR | p289/p290, qRT-PCR COG2F/COG2R (GII), probe RING2 | F | Farms, Abattoirs | N | [32] |
| Venezuela | Pigs | calicivirus | none | 0/137 (0) | RT-PCR | p289/p290, GLPSG1/YGDDI, GLPSG2/YGDD1 | F | Farms #7 | J +/- | [39] |
| | Cattle | GIII | GIII | 1/129 (1) | RT-PCR | NA | F | NA | NA | [56][12] |
| Argentina | Cattle | GIII | GIII.1, GIII.2, Not typed | 3/90 (3) | RT-PCR | CBECu-F/CBECu-R | F | Farms | J+ | [57] |
| Brazil | Pigs | GII.11/18/19 | GII.11 | 58/112 (52) | RT-PCR | SwNV1/SwNV2 | F | Farms | A- | [44] |
| | Pigs | calicivirus | GII.18 | 1/96 (1) | RT-PCR | p289/p289HI, p290/290HIJK, Mon431/432, 433/434 | RS | Farms #5 | J/A +/- | [58] |
| | Pigs | calicivirus | GII.11/18/19 | 20/261 (7) | RT-PCR | p289/p289HI, p290/p290HIJK, Cap C/D3/D1 | F/IC | Farms #6, Abattoirs #1 | J/A +/- | [59] |

| | | | | | | | | | | |
|---|---|---|---|---|---|---|---|---|---|---|
| | Pigs | calicivirus | none | 0/169 (0) | RT-PCR | p289/p290 | RS | Farms #5# | J+/- | [60] |
| | Pigs | calicivirus | GII.11 | 2/30 (7) | RT-PCR | p289/p290, SwNV1/SwNV2 | RS[c] | Farms #4# | J+ | [61] |
| | Cats | calicivirus | GIV.2 | 1/29 (3) | RT-PCR, Real-time RT-PCR | p289H/p290H, FNoV-09/FNoV-R15, Mon4FR, Probe Ring4 | F[c] | Shelters #4 | J+ | [62] |
| **Europe** | | | | | | | | | | |
| Italy | Pigs | GII.11/18/19 | none | 0/242 (0) | RT-PCR | PNV7/PNV8 | F | Farms #8 | J/A +/- | [76] |
| | Pigs | calicivirus | GII.11 | 1/201 (0,5) | RT-PCR | p290/p289/p110, PNV7/PNV8 | F | Farms #15 | J/A - | [77] |
| | | | none | 0/89 (0) | | | F | Farms #2 | J+ | |
| | Cattle | GIII | GIII.1/2 | 11/104 (11) | RT-PCR | CBECU-F/R | | Farms #16 | J- | [78] |
| | Cattle | GIII | GIII.1/2 | 21/101 (21) | RT-PCR | JV12/13 J11 U/L, J21 U/L, BEC-POL NBU-F/R, CBECU-F/R, J11U/CBECU-R | F | Farms | J- | [79] |
| | Dogs | GIV.2, GVI.2 | total | 66/516 (5) | ELISA | VLPS of GIV.2 (EF450827), GVI.2 (JF930689) | S | Households | J/A | [80] |
| | | | GIV.2 | 20/516 (4) | | | | | | |
| | | | GVI.2 | 46/516 (9) | | | | | | |
| | Dogs | calicivirus | GIV.2 | 4/183 (2) | RT-PCR | p289/p290, JV12Y/JV13I | | NA | J+ | [82] |
| | Dogs | GIV.2 | GIV.2 | 5/103 (5) | ELISA | VLP of GIV.2 (EF450827) | S | NA | A | [81] |
| | Dogs | GVI.2 | GVI.2 | 6/10 (60) | ELISA | VLPs of GVI.2 (GQ443611) | S | Clinics | J/A | [47] |
| | Dogs | calicivirus | GVI.2 | 11/239 (5) | RT-PCR | p298/p290, JV12Y/JV13I | F | Households | J+/- | [83] |
| | Dogs | calicivirus | GIV.2 | 1/1 (100) | RT-PCR | p289/p290 | F | NA | J+ | [192] |
| | Lion | calicivirus | GIV.2 | 1/1 (100) | real-time RT-PCR, RT-PCR | p290/p289, JV12Y/JV13I | F | Zoo #1 | J+ | [84] |
| | Cats | calicivirus | GIV.2 | 3/105 (3) | RT-PCR | p289/p290, JV12Y/JV13I | F | Shelters #3 | J+/- | [85] |
| | Cats | GIV.2 | GIV.2 | 34/211 (16) | ELISA | VLP of GIV.2 (EF450827) | F | Clinics, Shelter, outdoors | A | [81] |
| Spain | Pigs | calicivirus | NA | 27/221 (12) | real-time RT-PCR, RT-PCR | p289/p290, JV12/JV13. qRT PCR: probes for GI and GII | F | Farms #14, Abattoirs #1 | J/A+/- | [86] |
| | Dogs | calicivirus | GIV.2 | 2/26 (8) | RT-PCR | p298/p290, JV12Y/JV13I | F | Households | J+/- | [83] |
| | Dogs | Canine | GIV.2 | 7/7 (100) | RT-PCR | JV102/JV103 | F[c] | Kennel #1 | J+ | [88] |

| | | | | | | | | | | |
|---|---|---|---|---|---|---|---|---|---|---|
| Portugal | | Norovirus | | | | | | | | |
| | Dogs | calicivirus | GVI.2 | 29/105 (28) | RT-PCR | p289/p290, JV12Y/13I JV102/JV103 | F | Shelters, shops, clinics | +/- | [89] |
| | Dogs | Canine norovirus | GIV.2 | 60/256 (23) | RT-PCR | JV102/JV103 | F | shelter, shops clinic | +/- | [87] |
| | Dogs | GVI.2 | GVI.2 | 64/100 (64) | ELISA | VLPs of GVI.2 (GQ443611) | S | Clinics | J/A | [47] |
| Greece | Dogs | calicivirus | GIV.2 | 6/72 (8) | RT-PCR | p289/p290, JV12Y/JV13I | F[c] | Kennels, pet shop, clinic | J+ | [90] |
| UK | Cattle | GIII | GIII.1, | 77/100 (77) | ELISA | VLPs of GIII.1 (AJ011099), GIII.2 (AF320625) | S | Farms #5 | A | [93] |
| | | | GIII.2 | 87/100 (87) | | | | | | |
| | Cattle | | GIII.1 | 98/100 (98) | | | S | Farms #7 | J | |
| | | | GIII.2 | 66/100 (66) | | | | | | |
| | Cattle | NA | GIII | 44/398 (11) | Meta analysis | NA | F | Diagnostic | NA | [104] |
| | Dogs | calicivirus | none | 0/227 (0) | Real-time RT-PCR | Primers and probes based on GIV.2, GVI.1, GVI.2 | F[b] | Clinics #4, Kennels | J/A+/- | [105] |
| | | GIV.2, GVI.1/2 | total | 189/396 (48) | ELISA | VLPs of GIV.2 (EU224456), GVI.2 (GQ443611), GVI.1 (FJ692501) | S[b] | Kennel #1 Research Institut #1 | NA | |
| | | | GIV.2 | 47/396 (12) | | | | | | |
| | | | GVI.1 | 78/396 (20) | | | | | | |
| | | | GVI.2 | 8/396 (2) | | | | | | |
| | Dogs | canine norovirus | none | 0/248 (0) | Real-time RT-PCR | Canine specific primers and probes | F[b] | Clinics #6, Shelter #1 | J/A+/- | [106] |
| | | GIV.2, GVI.1, GVI.2 | GIV.2, GVI.1, GVI.2 | 147/325 (45) | ELISA | VLPs of GIV.2 (EU224456), GVI.2 (GQ443611), GVI.1 (FJ692501) | S[b] | Kennel #1 veterinary college #1 | NA | |
| | Dogs | GVI.2 | GVI.2 | 23/50 (46) | ELISA | VLPs of GVI.2 (GQ443611) | S | Clinics | J/A | [47] |
| | Mice[4] | norovirus | GV | 57/192 (67) | RT-PCR | GV specific, degenerate primers | F/RS[6] | Indoors | NA | [107] |
| | Mice[5] | | | 11/51 (22) | | | F[6] | Outdoors | NA | |
| Ireland | Pigs | calicivirus | none | 0/292 (0) | RT-PCR | p289/p290 | F | Farms #4 | J- | [40] |
| | Dogs | GVI.2 | none | 0/10 (0) | ELISA | VLPs of GVI.2 (GQ443611) | S | Clinics | J/A | [47] |
| Nether-lands | Pigs | norovirus | GII.11 | 2/100 (2) | RT-PCR, Southern blot | JV12/JV13 | PF | Farms #100 | A | [9] |
| | Cattle | | none | 0/43 (0) | | | PF | Farms #75 | J | |
| | Cattle | | GIII.2 | 25/75 (44) | | | PF | Farms #43 | A | |

| | | | | | | | | | | |
|---|---|---|---|---|---|---|---|---|---|---|
| | Chicken | | none | 0/48 (0) | | | PF | Broilers #48 | J | |
| | Cattle | norovirus | GIII.2 | 77/243 (32) | RT-PCR, EM, Southern blot, | JV12/JV13 | PF | Farms | J | [97] |
| | Cattle | norovirus | GIII.2 | 13/312 (4) | | | F[a] | Farms #1 | J+/- | |
| | Dogs | GVI.2 | GVI.2 | 17/50 (34) | ELISA | VLPs of GVI.2 (GQ443611) | S | Clinics | J/A | [47] |
| | Porpoise | HPNV | NA | 5/49 (10) | Real-time RT-PCR | HPNV primers, GII probe | IC[7] | NA | NA | [98] |
| | | | | 8/34 (24) | ELISA | VLPs of KP987888 | S | | | |
| Belgium | Pigs | calicivirus | GII.19 | 2/43 (5) | RT-PCR | JV12/JV13, p289/p290, swNo F/R | F | Diagnostic #1 | J/A+/- | [99] |
| | Cattle | calicivirus | GIII.2 | 10/133 (8) | RT-PCR | p298/p290, CBECu , BEC, JV12/JV13 | F[a] | Diagnostic #1 | J+ | [100] |
| | | GIII.2 | GIII.2 | 409/439 (93) | ELISA | VLPs of GIII.2 (EU794907) | S[b] | | J/A | |
| | Cattle | norovirus | GIII.2 | 15/317 (5) | RT-PCR | JV12/JV13 (all NV), CCV3/CCV4, CBECU-F/CBECU-R | F | Farms | NA | [193] |
| | Cattle | GIII | GIII.1, GIII.2 | 28/300 (9) | RT-PCR | CBeCu-F/R (bovine NoV), AMG1-F/R (based on GIII.1) sequence | F | Diagnostic #1 | J+ | [102] |
| | Cattle | GIII | GIII.2 | NA | RT-PCR | CBeCu-F/R | F | Diagnostic | J | [101] |
| Hungary | Pigs | calicivirus | GII.11 | 1/17 (6) | RT-PCR | p290/p289 | F | Farms #2 | J/A+/- | [112] |
| | Dogs | calicivirus | GVI | 2/63 (3) | RT-PCR | p290/p289 | F | Shelter | NA | [113] |
| | Dogs | GIV.2 | none | 0/10 (0) | ELISA | VLPs of GIV.2 (GQ443611) | S | Clinics | J/A | [47] |
| | Lab mice | calicivirus | GV | 10/41 (24) | RT-PCR | p289/p290, MNV specific primer set | PF | Outdoors | NA | [114] |
| | Mice[8] | | GV | 2/3 (67) | | | | | | |
| | Mice[9] | | none | 0/7 (0) | | | | | | |
| | Bank vole | | none | 0/3 (0) | | | | | | |
| Slovenia | Pigs | calicivirus | GII.11/18 | 5/406 (1) | RT-PCR | p290/NVp110, JV12Y/JV13I, JV12Y/NVp110 G1SKF/R, G2SKF/R | F | Farms #8 | J/A- | [42] |
| | Cattle | | GIII.2 | 2/108 (2) | | | F | Farms #4 | J/A- | |
| Poland | Dogs | GVI.2 | GVI.2 | 16/50 (32) | ELISA | VLPs of GVI.2 (GQ443611) | S | Clinics | J/A | [47] |

| | | | | | | | | | | |
|---|---|---|---|---|---|---|---|---|---|---|
| Finland | Dogs | GI, GII, GIV | none | 0/92 (0) | real-time RT-PCR | COG2R/QNIF2 QNIFS for GII, in house primers and QNIF4/QNIF3 for GI, Mon4F/Mon4R and Ring4 for GIV | F | Household | J/A+/- | [110] |
| | Dogs | GVI.2 | GVI.2 | 7/10 (70) | ELISA | VLPs of GVI.2 (GQ443611) | S | Clinics | J/A | [47] |
| Norway | Cattle | GIII | GIII.1, GIII.2 | 208/419 (50) | sybr green RT-PCR | BoNoV72F/BoNoV72R 2 CBECU-F/BoNoV72R2 | F | Farms #190 | J+/- | [109] |
| | Dogs | GVI.2 | GVI.2 | 16/50 (32) | ELISA | VLPs of GVI.2 (GQ443611) | S | Clinics | J/A | [47] |
| Sweden | Dogs | GVI.2 | GVI.2 | 4/10 (40) | ELISA | VLPs of GVI.2 (GQ443611) | S | Clinics | J/A | [47] |
| Denmark | Rat[10] | calicivirus | none | 0/11 (0) | RT-PCR | p289CVa/p289CVb, p289CVc/p289CVd | IC | Sewer system #5 | NA | [108] |
| | Dogs | GVI.2 | GVI.2 | 10/50 (20) | ELISA | VLPs of GVI.2 (GQ443611) | S | Clinics | J/A | [47] |
| Germany | Pigs | GI, GII | GII.18 | 17/120 (14) | RT-PCR | Mon431-434 | F | Abattoirs #3 | A | [41] |
| | Cattle | GIII | GIII.1 | 66/100 (66) | ELISA | VLPs of GIII.1 (AJ011099), GIII.2 (AF320625) | S | Farms #12 | A | [93] |
| | | | GIII.2 | 87/100 (87) | | | | | | |
| | Cattle | | GIII.1 | 71/100 (71) | | | S | Farms #6 | J | |
| | | | GIII.2 | 94/100 (94) | | | | | | |
| | Cattle | norovirus | GIII.2 | 409/439 (93) | RT-PCR | NV32/NV36, NV33/NV35, JV12/JV13 BECF,BECR | F | Farms #29 | J/A+ | [95] |
| | Cattle | GIII.1 | GIII.1 | 817/824 (99) | ELISA | VLPs of GIII.1 (AJ011099) | S | Farms #25 | J/A | [94] |
| | | | | 34/381 (9) | antigen ELISA, | Anti-GIII.1 VLP hyperimune sera | F | Farms | + | |
| | Dogs | GVI.2 | GVI.2 | 8/50 (16) | ELISA | VLPs of GVI.2 (GQ443611) | S | Clinics | J/A | [47] |
| | Dogs | all | GIV.2 | 13/294 (4) | RT-PCR | p298/p290, JV12Y/JV13I | F | Households | J+/- | [83] |
| | Rat[10] | all | GV | 2/20 (10) | NGS | | IC | Outdoors | NA | [96] |
| Switzerland | Dogs | GVI.2 | GVI.2 | 2/10 (20) | ELISA | VLPs of GVI.2 (GQ443611) | S | Clinics | J/A | [47] |
| France | Dogs | GVI.2 | GVI.2 | 10/50 (20) | ELISA | VLPs of GVI.2 (GQ443611) | S | Clinics | J/A | [47] |
| | Dogs | calicivirus | none | 0/26 (0) | RT-PCR | p298/p290, JV12Y/JV13I | F | Households | J+/- | [83] |
| | Cattle | GIII | GIII.1/2 | 89/456 (20) | RT-PCR | CBECU-F/CBECU-R | F[c] | Farms #415 | J+ | [91] |

| | | | | | | | | | | |
|---|---|---|---|---|---|---|---|---|---|---|
| | Cattle | GIII | GIII.1/2 | 29/81 (36) | RT-PCR | CBECU-F/CBECU-R | F[b] | Farms NA | J+ | [92] |
| **Africa** | | | | | | | | | | |
| South Africa | Pigs | calicivirus | none | 0/120 (0) | RT-PCR | p290/p289 | RS | Farms #2 | J | [117] |
| Ethiopia | Pigs | calicivirus | none | 0/117 (0) | RT-PCR | p290/p110, G2SKF/G2SKR | F | Households | J/A | [43] |
| Egypt | Cattle | GIII | GIII.2 | 6/25 (24) | RT-PCR | CBECU-F/CBECU-R | F | Farms #2 | J+ | [115] |
| Tunisia | Cattle | GIII | GIII.2 | 28/169 (17) | RT-PCR | CBECU-F/CBECU-R | F | Farms #17 | J+ | [116] |
| **Asia (including Australia/New Zealand)** | | | | | | | | | | |
| China | Pigs | calicivirus | none | 0/209 (0) | RT-PCR | p289/p290 | F | Farms #6 | J/A- | [33] |
| | Pigs | GII.11/18/19 | GII.11 -NA | 2/12 (17) | RT-PCR | not stated | RS | Farms #3 | J+ | [26, 27] |
| | Pigs | GII.11 | GII.11 | 2/904 (0,2) | RT-PCR | p289/p290 | F | Farms #14 | J/A | [35] |
| | Dogs | NA | GVII | 2/NA (NA) | NA | NA | RS | NA | NA | [194] |
| | Cattle | Bovine norovirus | GIII.1 | 3/28 (11) | RT-PCR | CBECU-F/CBECU-R | F | Farms #5 | J+ | [63] |
| | Bats | calicivirus | Not typed | NA | metagenomics | NA | RS | Outdoors | NA | [130] |
| | Bats | Not typed | Not typed | 2/62 (3) | RT-PCR | Primers designed based on newly found virus | RS | Outdoors | A- | [64] |
| | Bats | norovirus | NA | 10/235 (4)** | metagenomics | Primers designed based on newly found virus | IC | Outdoors | A- | [6] |
| | Brown rat | NA | GV | NA | RT-PCR | NA | RS | NA | NA | [195] |
| Taiwan | Pigs | calicivirus | GII.11 | 9/533 (2) | RT-PCR | p290/p110, NV2oF2/NV2oR, G2F3/G2SKR | F | Farms #6 | J/A- | [34] |
| Japan | Pigs | GII.11 | | 95/266 (36) | ELISA | VLPs of GII.11 (SW918) | S | NA | J | [46] |
| | Pigs | calicivirus | GII.11/18/19 | 36/240 (15) | RT-PCR, real-time RT-PCR | p289/p290, G1SKF/G1SKR, G2SKF/G2SKR qRT-PCR: COG2F/ALPF/COG2R | IC[a] | Farms #3 Abattoirs #1 | A- | [36] |
| | Pigs | norovirus | GII.11 | 4/1117 (0.4) | RT-PCR | 35/36 (SRSV), NV81/NV82 | IC[3] | Farms #26 | - | [10] |
| | Pigs | calicivirus | GII.11 | 1/24 (4) | RT-PCR | p289/p290 | IC | Diagnostic lab #1 | J+/- | [45] |

| | | | | | | | | | | |
|---|---|---|---|---|---|---|---|---|---|---|
| | Dogs | calicivirus | GIV (NA) | 2/97 (2) | RT-PCR | p290d/p289d | F | Households | + | [65] |
| | Cats | | GIV.2 | 1/83 (1) | | | | | | |
| | Cats | caliciviurs | GIV.2 | 1/NA (NA) | RT-PCR | p290d/p289d | RS | Shelter | - | [127] |
| | Mice [11] | GV | GV | 6/44 (14) | RT-PCR | MNV-F1/MNV-R1, MNV-F2/MNV-R2 | IC | Outdoors | NA | [66] |
| | Rat[12] | | GV | 1/1 (100) | | | | | | |
| | Grass vole[5] | | none | 0/1 (0) | | | | | | |
| Korea | Pigs | norovirus | GII.11/18 | 10/537 (2) | RT-PCR | GIIF2/GIIR, GIIF1/GIIF2, GIIF2/GIIR | F | Farms #64 | J/A +/- | [67] |
| | Pigs | norovirus | GII.11/18 | 3/567 (0,5) | RT-PCR | NORO-DG35OF/IF | F | Farms #12 | NA | [37] |
| | Dogs | Canine norovirus | GIV.2 | 14/459 (3) | RT-PCR | JV102/JV103 | F/S | Clinic, Shelters | +/- | [68] |
| | | | GVI.2 | 68/427 (16) | ELISA | ELISA: p-domain of GVI.2 (GQ443611) | | | | |
| | Cattle | GIII | GIII.1/2 | 60/645 (9) | RT-PCR | bovine norovirus-specific primers | F | Farms #629 | J+ | [69] |
| India | Cattle | GIII | GIII.1 | 1/249 (0,4) | RT-PCR | BEC-POL NBU-F/R CBECU-F/R, J11U/CBECU-R | F | NA | + | [13] |
| Iran | Cattle | GIII | GIII.1/2 | 100/253 (40) | RT-PCR | CBECU-F/CBECU-R | F[a] | Farms #42 | J+ | [70] |
| | Cattle | GI, GII | GIII | 9/50 (18) | RT-PCR | Ni/E3 | F | Farms #6 | + | [71] |
| Turkey | Cattle | GIII | GIII.2 | 6/70 (9) | real-time RT-PCR | IIIBoVo-F/R | F | Farms | J+ | [72] |
| | Cattle | GIII | GIII.2 | 5/127 (4) | RT-PCR | BoNoV851-F/BoNoV1350-R | RS | NA | J+ | [73] |
| New Zealand | Pigs | GI-GIII | GII.11 | 2/23 (9) | RT-PCR | primers that detects GI-GIII | F | Farms #2 | J- | [38] |
| | Cattle | GI-GIII | GIII.1 | 15/28 (54) | real-time RT-PCR | newly designed based on 52 NoV sequences | F | Farms #2 | - | [75] |
| | Sheep | GI-GIII | GIII.3 | 8/33 (24) | RT-PCR | primers that detects GI-GIII | F | Farms #2 | J- | [38] |
| Australia | Cattle | calicivirus | GIII.2 | 2/8 (25) | RT-PCR | p289/p290, CBECU-F/CBECU-R | PF | Farms | J+ | [196] |

[1] 64/312 (20%) in finisher pigs, 36/176 (20%) farms

[2] farms taken as n

[3] Caecum content

[4] House mouse (*Mus musculus*)

[5] Wood mouse (*Apodemus sylvaticus*)

[6] From carcass

[7] RNA from FFPE porpoise intestinal tissues

[8] Striped field mice (*Apodemus agrarius*)

[9] Yellow - necked mice (*Apodemus flavicollis*)

[10] Norway rat (*Rattus Norvegicus*)

[11] Japanese field mouse (*Apodemus speciosus*)

[12] Black rat (*Rattus rattus*)

[*] Used same set of veal calfs'

[**] 9/45 (20) and 1/44 (2,3) in two different region

**Supplementary Table 2**. Studies investigating human-to-animal transmission and the detection methods that were applied. Included are studies that analysed faecal samples for human norovirus sequences by RT-PCR or real-time RT-PCR or serological studies. Primers most often used are the universal calicivirus primer sets Some of the most frequently used primers are p289/p290 and p290/p110 (universal calicivirus primers, RdRp) and JV12/JV13 (norovirus specific). ELISA refers to serology ELISA. Unless otherwise stated sample were collected at one time point. a=longitudinal study, b=case-control, c=outbreak
A=adult (in pigs >6month), J=juvenile

| Location | Host | Investigated genogroup/ Genotype | Human norovirus detected | Prevalence (%) | Detection method | Primers, Probes and antigens used | Sample collection | | Age Clinical status Adult (A) Juvenile (J) Symptomatic (+) Asymptomatic (-) | Ref |
|---|---|---|---|---|---|---|---|---|---|---|
| | | | | | | | Sample type Faeces (F) Pooled Faeces (PF) Sera (S) Intestinal content (IC) | Locations (#) | | |
| **The Americas** | | | | | | | | | | |
| **USA** | Pigs | GI.1, GII.4 | GI.1 | 57/110 (63) | ELISA | VLPS of GI.1, GII.1 | S | Farms Abattoirs #1 | NA | [46] |
| | | | GII.4 | 47/110 (52) | | | | | | |
| | | calicivirus | none | 0/117 (0) | RT-PCR | P289H,I/p290H,I,J,K | F/IC | Farms #1 Abattoirs #2 | J- | |
| | | GI, GII | NA | 2/104 | Antigen ELISA | Hyperimmune sera against GI.1, GI.2, GI.3, GII.1, GII.3, GII.4 and GII.9 | F | Farms #1 | J- | |
| | Pigs | calicivirus | none | 0/9 (0)[1] | RT-PCR | p290/p110, G2SKF/G2SKR | PF | Farms #9 | A- | [28] |
| | Pigs | calicivirus | none | 0/275 (0) | RT-PCR | p290/p110 | F | Farms #6 Abattoirs #1 | A- | [25] |
| | Pigs | calicivirus | none | 0/139 (0) | RT-PCR | p290/p110 | F | Farms #3 | J | [48] |
| | Capuchin | | none | 0/5 (0) | ELISA | VLPs of GI.1, GII.4, GII.7 | S | | J/A- | [140] |
| | | | total | 33/39 (85) | | | | | | |

| | | | | | | | | | | |
|---|---|---|---|---|---|---|---|---|---|---|
| | Mangabey | GI.1, GII.4, GII.7 | GI.1 | 29/39 (74) | | | | Research institute #1 | | |
| | | | GII.4 | 28/39 (72) | | | | | | |
| | | | GII.7 | 15/39 (38) | | | | | | |
| | Pigtail | | total | 11/17 (65) | | | | | | |
| | | | GI.1 | 4/17 (24) | | | | | | |
| | | | GII.4 | 4/17 (24) | | | | | | |
| | | | GII.7 | 6/17 (35) | | | | | | |
| | Rhesus | | total | 17/27 (63) | | | | | | |
| | | | GI.1 | 11/27 (41) | | | | | | |
| | | | GII.4 | 8/27 (30) | | | | | | |
| | | | GII.7 | 4/27 (15) | | | | | | |
| | Chimp | | total | 11/12 (92) | | | | | | |
| | | | GI.1 | 11/12 (92) | | | | | | |
| | | | GII.4 | 2/12 (17) | | | | | | |
| | | | GII.7 | 0/12 (0) | | | | | | |
| | Rhesus | calicivirus | Total | 41/500 (8) | Real-time RT-PCR | Primers/probes specific for GI, GII, GIV | RS | Research institute #1 | J+/- | [138, 139] |
| | | | GI.1 | 30/500 (6) | | | | | | |
| | | | GII.7 | 8/500 (1,6) | | | | | | |
| | | | GIV | 3/500 (0,6) | | | | | | |
| | Rhesus | GI.1 | GI.1 | 114/188 (61) | ELISA | VLPs of GI.1, GII.5 | S[a] | Research institute #1 | J/A | [141] |
| | | GII.5 | GII.5 | 97/188 (52) | | | | | | |
| | Cats | calicivirus | none | 0/24 (0) | RT-PCR | p289/p290 | F | Clinic #1 Shelter #1 | J/A+/- | [53] |
| **Canada** | Pigs | calicivirus | none | 0/20 (0) | RT-PCR | p289/p290 | PF/F[2] | Farms #20, Abattoirs #2 | J/A | [31] |
| | Pigs | norovirus | GII.4 | 4/120 (3) | RT-PCR | Region B and A primers, MON431-MON434, G1/G2 | PF[a] | Farms #10 | NA | [30] |
| | Cattle | | | 1/179 (0,5) | | | PF | Farms #45 | J/A | |
| **Venezuela** | Pigs | calicivirus | none | 0/137 (0) | RT-PCR | p289/p290, GLPSG1/YGDDI1 GLPSG2/YGDD1 | F | Farms #7 | J +/- | [39] |
| **Nicaragua** | Pigs | GII.4 Dijon | GII.4 Dijon | 96/137 (70) | ELISA | VLPs of GII.4 ( GU325839, AF472623), GII.3 (AY247431)[3] | S | Households | J/A | [133] |
| | | GII.4 HS194 | GII.4 HS194 | 94/137 (69) | | | | | | |
| | | GII.3 | GII.3 | 80/137 (58) | | | | | | |

| | | | | | | | | | | |
|---|---|---|---|---|---|---|---|---|---|---|
| **Brazil** | Pigs | calicivirus | none | 0/96 (0) | RT-PCR | p289/289HI, p290/290HIJK, Mon431/432/433/434 | RS | Farms #5 | J/A +/- | [58] |
| | Pigs | calicivirus | none | 0/261 (0) | RT-PCR | 289/289HI, 290/290HIJK, Cap C/D3/D1 | F/IC | Farms #6, Abattoirs #1 | J/A +/- | [59] |
| | Pigs | calicivirus | none | 0/169 (0) | RT-PCR | p289/p290 | RS | Farms #5# | J+/- | [60] |
| | Primates[4] | calicivirus | none | 0/62 (0) | RT-PCR | p289/p290 | F | Outdoors | - | [197] |
| | Cats | calicivirus | none | 0/29 (0) | Real-time RT-PCR | p289H/p290H, FNOV-09/FNOV-R15 | F | Shelters #4 | J+ | [62] |
| **Europe** | | | | | | | | | | |
| **Italy** | Pigs | calicivirus | none | 0/290 (0) | RT-PCR | p290/p289/p110 | F | Farms #17 | J/A +/- | [77] |
| | dogs | calicivirus | GIV.2 | 0/183 (0) | RT-PCR | p289/p290, JV12Y/JV13I | F | NA | J+ | [82] |
| | Dogs | GII.4 | GII.4 | 52/516 (10) | ELISA | VLPS of GII.4 (AY032605), GIV.1 (AF414427) | S | Households | J/A | [80] |
| | | GIV.1 | GIV.1 | 3/516 (0,6) | | | | | | |
| | Dogs | calicivirus | none | 0/239 (0) | RT-PCR | p298/p290, JV12/JV13 | F | Households | J+/- | [83] |
| | Cats | calicivirus | none | 0/105 (0) | RT-PCR | p298/p290, JV12/JV13 | F | Shelters #3 | J+/- | [85] |
| **Spain** | Pigs | calicivirus | NA | 0/221 (0) | Real-time RT-PCR, RT-PCR | p289/p290, JV12/JV13, For real-time RT-PCR probes for GI and GII | F | Farms #14, Abattoirs #1 | J/A+/- | [86] |
| | Dogs | calicivirus | none | 0/26 (0) | RT-PCR | p298/p290, JV12/JV13 | F | Households | J+/- | [83] |
| **Portugal** | Dogs | calicivirus | none | 0/105 (0) | RT-PCR | p298/p290, JV12/JV13 | F | Shelters, shops, clinics | +/- | [89] |
| **UK** | Cattle | GI, GII | none | 0/476 (0) | RT-PCR | NI/E3 (GII), GLPSG1/YGDD1 GLPSG2/YGDD1 | F | Farms | J/A+ | [198] |
| | Dogs | calicivirus | none | 0/227 (0) | SYBR-based qPCR | p289/p290 | F[b] | Clinics #4, Kennels | J/A+/- | [105] |

| | | | | | | | | | | |
|---|---|---|---|---|---|---|---|---|---|---|
| | Dogs | GI, GII | none | 0/248 (0) | Real-time RT-PCR | Primers to detect GI, GII | F[b] | Clinics #6, Shelter #1 | J/A+/- | [106] |
| | Dogs | GI, GII | total | 43/325 (13) | ELISA | Pooled VLPs for GI: GI.1, GI.2, GI.3 GII: GII.3, GII.4, GII.6, GII.12 | S[b] | Kennel #1 veterinary college #1 | NA | [106] |
| | | GI | GI.1/2/3 | 8/325 (3) | | | | | | |
| | | GII | GII.3/4/6/ 12 | 20/325 (6) | | | | | | |
| | Mice[5] | Norovirus | none | 0/92 (0) | RT-PCR | general norovirus primers | F/RS[6] | Outdoors | NA | [107] |
| **Ireland** | Pigs | calicivirus | none | 0/292 (0) | RT-PCR | p289/p290 | F | Farms #4 | J- | [40] |
| **Netherlands** | Pigs | norovirus | none | 0/100 (0) | RT-PCR, Southern blot | primers designed based on human norovirus | PF | Farms #100 | A | [9] |
| | Cattle | norovirus | none | 0/118 (0) | | | PF | Farms #118 | J/A | |
| | Primates[7] | GI, GII | none | 0/201 (0) | RT-PCR, | JV12/JV13 | F | Research Institute #1 | +/- | [142] |
| | | | | 0/158 (0) | ELISA | VLPs of GI.1, GII.4 (1995) | S | Research institute #1 | NA | [142] |
| | Chicken | norovirus | none | 0/48 (0) | RT-PCR, Southern blot | primers designed based on human norovirus | PF | Broilers #48 | J | [9] |
| **Belgium** | Pigs | calicivirus | none | 0/43 (0) | RT-PCR | JV12/JV13, p289/p290 | F | Diagnostic #1 | J/A+/- | [99] |
| | Cattle | calicivirus | none | 0/133 (0) | RT-PCR | JV12/JV13, p298/p290 | F[a] | Diagnostic #1 | J+ | [100] |
| | Cattle | Norovirus | none | 0/317 (0) | RT-PCR | JV12/JV13, CCV3/CCV4, CBECU-F/CBECU-R | F[a] | Farms | J/A+/- | [193] |
| **Hungary** | Pigs | calicivirus | none | 0/17 (0) | RT-PCR | p289/p290 | F | Farms #2 | J/A+/- | [112] |
| | Dogs | caliciviurs | none | 0/63 (0) | RT-PCR | p289/p290 | F | Shelter | | [113] |
| | Rodents[8] | calicivirus | none | 0/54 (0) | RT-PCR | p289/p290 | PF | Outdoors | NA | [114] |
| **Slovenia** | Pigs | calicivirus | none | 0/406 (0) | RT-PCR | p290/NVp110, JV12Y/JV13I | F | Farms #8 | J/A- | [42] |
| | Cattle | | | 0/119 (0) | | | F | Farms #4 | J/A- | |

| | | | | | | | | | | |
|---|---|---|---|---|---|---|---|---|---|---|
| | | | | | | JV12Y/NVp110 G1SKF/R G2SKF/R | | | | |
| **Finland** | Dogs | GI, GII, GIV | GII.4 | 3/92 (3) | Real-time RT-PCR | COG2R/QNIF2 QNIFS for GII, in house primers and QNIF4/QNIF3 for GI, Mon4F/Mon4R and Ring4 for GIV | F | Household | J/A+/- | [110] |
| | | | GII.12 | 1/92 (1) | | | | | | |
| | Rats[9] | GI, GII | NA | 2/100 (2) | Real-time RT-PCR | GII: COG2R/QNIF2 and QNIFS (probe) GI: QNIF4/QNIF3 and JJV1P (probe) | F/IC[6] | Dump sites #3 | NA | [111] |
| | Mice[10] | | | 0/88 (0) | | | | Outdoors | | |
| | Birds | GI, GII | Total | 31/115 (27) | | | F[12] | Dump sites #1 | | |
| | | | GII.4 | 4/115 (4) | | | | | | |
| | | | GII.3 | 2/115 (2) | | | | | | |
| | | | GII[11] | 19/115 (17) | | | | | | |
| | | | GI[11] | 6/115 (5) | | | | | | |
| **Denmark** | Rats[8] | calicivirus | GI.pb-GI.6 | 1/11 (9) | Real-time RT-PCR IEM | p289CVa/p289CVb p289CVc/p289CVd | IC[6] | Sewersystem #5 | NA | [108] |
| **Germany** | Dogs | caliciviurs | none | 0/3 (0) | RT-PCR | p298/p290, JV12Y/JV13I | F | Households | J+/- | [83] |
| | Norway rat[11] | all | GV | 0/20 (0) | NGS | | IC | outdoors | NA | [96] |
| **France** | Dogs | calicivirus | none | 0/26 (0) | RT-PCR | p298/p290, JV12Y/JV13I | F | Households | J+/- | [83] |
| **Europe** | Dogs | GI.1, GII.4 | GI.1 | 9/308 (3) | ELISA | VLPs of GI.1 (NC_001959), GII.4 (JQ478409.1) | S | Clinics | J/A | [47] |
| | | | GII.4 | 106/308 (34) | | | | | | |
| **Africa** | | | | | | | | | | |
| **South Africa** | Pigs | calicivirus | NA | 1/120 (1) | RT-PCR | p289/p290 | RS | Farms #2 | J | [117] |
| **Ethiopia** | Pigs | calicivirus | GII.1 | 2/117 (2) | RT-PCR | p290/p110, G2SKF/G2SKR | F | Households | J/A | [43] |
| **Asia (including Australia/New Zealand)** | | | | | | | | | | |
| **China** | Pigs | calicivirus | none | 0/209 (0) | RT-PCR | p289/p290 | F | Farms #6 | J/A- | [33] |
| | Pigs | calicivirus | none | 0/904 (0) | RT-PCR | p289/p290 | F | Farms #14 | J/A | [35] |

| | | | | | | | | | |
|---|---|---|---|---|---|---|---|---|---|
| | Macaques [13] | calicivirus | GII.17 | 16/50 (32) | RT-PCR | GII.17 specific primers | F | Farm #1 | NA | [136],[137] |
| **Taiwan** | Pigs | calicivirus | GII.2/4 | 82/533 (32) | RT-PCR | p290/p110, NV2oF2/NV2oR, G2F3/G2SKR | F | Farms #6 | J/A- | [34] |
| **Japan** | Pigs | calicivirus | Total | 11/354 (3) | RT-PCR, Real-time RT-PCR | p289/p290, G1SKF/G1SKR G2SKF/G2SKR . Real-time RT-PCR primers: COG2F/ALPF/COG2R | IC[a] | Farms #3 Abattoirs #1 | A- | [36] |
| | | | GII.3 | 4/240 | | | | | | |
| | | | GII.4 | 7/240 | | | | | | |
| | | | GII.13 | 1/240 | | | | | | |
| | Pigs | SRSV | none | 0/1117 (0) | RT-PCR | 35/36 (SRSV), NV81/NV82 | IC[2] | Farms #26 | - | [10] |
| | Pigs | calicivirus | none | 0/24 (0) | RT-PCR | p289/p290 | IC | Diagnostic #1 | J+/- | [45] |
| | Dogs | caliciviurs | none | 0/97 (0) | RT-PCR | p290d/p289d | F | Households | + | [65] |
| | Cats | | | 0/83 (0) | | | | | | |
| **Korea** | Pigs | GII | none | 0/537 (0) | RT-PCR | GIIF2/GIIR (GII) | F | Farms #64 | J/A +/- | [67] |
| | Pigs | Norovirus | none | 0/576 (0) | RT-PCR | NORO-DG35OF/IF | F | Farms #12 | NA | [37] |
| **Iran** | Cattle | GI, GII | NA | 9/50 (18) | RT-PCR | Ni/E3 | F | Farms #6 | + | [71] |
| **New Zealand** | Pigs | GI, GII | none | 0/23 (0) | RT-PCR | GI-GIII primers | F | Farms #2 | J- | [38] |
| | Sheep | | | 0/33 (0) | | | | Farms #2 | J- | |
| | Cattle | GI, GII | none | 15/28 (0) | real-time RT-PCR | newly designed based on 52 norovirus sequences | F | Farms #2 | - | [75] |
| **Australia** | Cattle | calicivirus | none | 0/6 (0) | RT-PCR | p289/p290 | PF | Farms | J- | [196] |

[1] Number of farms (n)

[2] Caecum content

[3] From chronic patient

[4] Red-howler (*Alouatta seniculus*), Commo marmosets (*Callithrix jacchus*, free-ranging), Black-faced lion tamarin (*Leontopithecus caissara*)

[5] *Mus musculus*

[6] From carcass

[7] Chimpanzees *(Pan troglodytes)*, Rhesus macaque (*Macaca mulatta*), Cotton top tamarins (*Saguinus Oedipus*), Cynomolgus macaque (*Macaca fascicularis*), Common marmoset

[8] Mice (*Apodemus agrarius*), Yellow necked mouse (*Apodemus flavicollis*), bank voles (*Myodes glareolus*)

[9] Norway rat *(Rattus norvegicus)*

[10] Yellow necked mouse (*Apodemus flavicollis*)

[11] Could not be sequenced

[12] Collected from untouched fresh snow

[13] Rhesus/Pigtail/cynomologus

**Supplementary Table 3.** Summary of VLP attachment studies. Shown are the putative norovirus hosts for which attachment studies have been conducted. Their HBGA was extracted from papers that were chosen for the review. No additional papers were included.

| Host | HBGA phenotype (determined from) | VLPs (accession number) | Binding detected | No binding detected | Ref |
|---|---|---|---|---|---|
| Human | A+/B+/H1+/H2+/H3+/ Lewa+/Lewb+/Lewx+/Lewy+ | GII.4 (AAK84679) GII.5 (AF397156) GII.9 (AY038599) | PGM, PGIT | NA | [199] |
| | | GI.1 | PGM, PGIT[3,4] Epithelial cells of pig intestinal tissue | NA | |
| | | GI.1 (JX023285) | Type II/III PGM[4] | NA | [200] |
| | | GI.1, GI.3, GII.1, GII.3, GII.4 | Porcine buccal and duodenal tissue[3] | GI.3 did not bind | [146] |
| | | GI.1 NC_001959.2) GII.4 (AF472623) | Canine saliva/duodenal scrapings of secretors Canine duodenal tissue (antigen-A independent) | Human saliva of non-secretors | [106] |
| | | GI.3 (KP064096) GII.3 (KP064097) GII.6 (KP064098) GII.12 (KP064099) | Canine duodenal scrapings of secretors | NA | [106] |
| | | GII.4 (Dijon 171/96) | Human saliva of secretors | Bovine erythrocytes | [177] |
| | | GII.4 (GU325839, AF472623), GII.3 (AY247431) | Type III PGM[3] | NA | [133] |
| | | GII.1 | NA | Bovine cell lines | [201] |
| | | GI.1 | Erythrocytes of chimpanzees | Erythrocytes of baboon, spider monkey, rhesus macaque, chicken, guinea pig, murine, canine, bovine, porcine, feline, and lapine | [202] |
| Pig | A+/H1+/H2+/Lewb+ (PGM/PGIT, tissue) | GII.11 | NA | 52 human saliva (representing all major HBGA types) | [46] |
| Cattle | A+/B-/H2+/Ley+/H1-/H3-/Lewa+[2] (fixed tissue) | GIII.2 (AF097917) | Bovine intestinal tissue (stomach, duodenum), bovine eryhthrocytes Bovine saliva Porcine intestinal tissue (non-epithelial cells) 2/44 synthetic glycans: (alpaGal-Lex, alphaGal tri, both have a terminal alpha 1,3 galactose | Human saliva (A/B/O types)/ intestinal tissue 42/44 synthetic glycans tested | [177] |
| | | GIII.2 (EU794907) | Bovine cell lines[1] | NA | [201] |

| | | | | | |
|---|---|---|---|---|---|
| Bat | NA | Bat norovirus (KJ790198) | H1, H3, A, B, 2,6-sialic acid (highest) (at 3 different temperatures) | H2, -2,3-sialic acid Lew a,b,x,y | [129] |
| Rhesus macaques | A+/B+/Lewb+/Lewx+/Lewy+/Lewa- (saliva) | GII.17 (KX356908) | Human saliva | NA | [136, 138, 139] |
| Dog | H2+/A+/B-/Lewy+/Lewa-/Lewb-/Lewx- (saliva, intestinal scraping) | GVI.2 (GQ443611) GVI.1 (FJ692501) GIV.2 (EU224456.1) | Canine tissue (antigen-A independent)[3,5] 4/20 synthetic glycans: H1, A hepta, Lewb, Sial LNF V (1,2 fucose) | 16/20 synthetic glycans | [176] |
| | | GVI.2 (GQ443611) | Canine/human saliva of secretors | Human saliva of non-secretors | [176] |
| Cats | NA | GIV.2 (JF781268) | NA | 30 synthetic glycans | [203] |

[1] Madine-darby kidney cells (MDBK), bovine turbinate cells, embryonic bovine tracheal cells, Georgia bovine kidney cells, embryonic bovine lung cells, Mac T-cells (udder origin), bronchial cells, Bomac cells (macrophagic origin), jejunocytes

[2] only in goblet cells of duodenum and colon

[3] Blocking experiments were carried out

[4] VLP binding was abolished with KIO4 pre-treatment (oxidizes carbohydrates)

[5] Pre-treatment with 1,2-alpha-fucosidase diminished VLP binding to tissue (removes the fucose group from the H antigen)

[6] Isolated from rhesus macaques

PGM = Pig intestinal mucin, PGIT = pig gastrointestinal tissue washing, NA=not applicable

**References**


1. Vinje, J., Advances in Laboratory Methods for Detection and Typing of Norovirus. *J Clin Microbiol* **2015,** 53, (2), 373-381.10.1128/JCM.01535-14
2. Throne, L.; Goodfellow, I., Norovirus gene expression and replication. *J Gen Virol* **2014,** 95, 278-291.10.1099/vir.0.059634-0
3. Bull, R. A.; Tanaka, M. M.; White, P. A., Norovirus recombination. *J Gen Virol* **2007,** 88, 3347–3359.10.1099/vir.0.83321-0
4. Bull, R. A.; Hansman, G. S.; Clancy, L. E.; Tanaka, M. M.; Rawlinson, W. D.; White, P. A., Norovirus Recombination in ORF1/ORF2 Overlap. *Emerg Infect Dis* **2005,** 11, (7), 1079-1085.10.3201/eid1107.041273
5. Kroneman, A.; Vega, E.; Vennema, H.; Vinjé, J.; White, P. A.; Hansman, G.; Green, K.; Martella, V.; Katayama, K.; Koopmans, M., Proposal for a unified norovirus nomenclature and genotyping. *Arch Virol* **2013,** 158, (10), 2059-2068.10.1007/s00705-013-1708-5
6. Hu, D.; Zhu, C.; Wang, Y.; Ai, L.; Yang, L.; Ye, F.; Ding, C.; Chen, J.; He, B.; Zhu, J.; Qian, H.; Xu, W.; Feng, Y.; Tan, W.; Wang, C., Virome analysis for identification of novel mammalian viruses in bats from Southeast China. *Sci. rep.* **2017,** 7, (1), 10917.10.1038/s41598-017-11384-w
7. Shi, M.; Lin, X. D.; Chen, X.; Tian, J. H.; Chen, L. J.; Li, K.; Wang, W.; Eden, J. S.; Shen, J. J.; Liu, L.; Holmes, E. C.; Zhang, Y. Z., The evolutionary history of vertebrate RNA viruses. *Nature* **2018,** 556, (7700), 197-202.10.1038/s41586-018-0012-7
8. Siebenga, J. J.; Vennema, H.; Zheng, D. P.; Vinjé, J.; Lee, B. E.; Pang, X. L.; Ho, E. C. M.; Lim, W.; Choudekar, A.; Broor, S.; Halperin, T.; Rasool, N.; Hewitt, J.; Greening, G.; Jin, M.; Duan, Z. J.; Lucero, Y.; O'Ryan, M.; Hoehne, M.; Schreier, E.; Ratcliff, R.; White, P.; Iritani, N.; Reuter, G.; Koopmans, M., Norovirus illness is a global problem: emergence and spread of norovirus GII. 4 variants, 2001–2007. *J Infect Dis* **2009,** 200, (5), 802-812.10.1086/605127
9. Van Der Poel, W. H. M.; Vinjé, J.; Van Der Heide, R.; Herrera, M. I.; Vivo, A.; Koopmans, M. P. G., Norwalk-like calicivirus genes in farm animals. *Emerg Infect Dis* **2000,** 6, (1), 36-41.10.3201/eid0601.000106

10. Sugieda, M.; Nagaoka, H.; Kakishima, Y.; Ohshita, T.; Nakamura, S.; Nakajima, S., Detection of Norwalk-like virus genes in the caecum contents of pigs. Brief report. *Arch Virol* **1998,** 143, (6), 1215-1221.10.1007/s007050050369
11. Dastjerdi, A. M.; Green, J.; Gallimore, C. I.; Brown, D. W. G.; Bridger, J. C., The bovine Newbury agent-2 is genetically more closely related to human SRSVs than to animal caliciviruses. *Virology* **1999,** 254, (1), 1-5.10.1006/viro.1998.9514
12. Liu, B. L.; Lambden, P. R.; Günther, H.; Otto, P.; Elschner, M.; Clarke, I. N., Molecular characterization of a bovine enteric calicivirus: Relationship to the Norwalk-like viruses. *J Virol* **1999,** 73, (1), 819-825PMC103897
13. Menon, V. K.; George, S.; Shanti, A. A.; Saravanabavan, A.; Samuel, P.; Ramani, S.; Estes, M. K.; Kang, G., Exposure to human and bovine noroviruses in a birth Cohort in southern India from 2002 to 2006. *J Clin Microbiol* **2013,** 51, (7), 2391-2395.10.1128/jcm.01015-13
14. Vildevall, M.; Grahn, A.; Oliver, S. L.; Bridger, J. C.; Charpilienne, A.; Poncet, D.; Larson, G.; Svensson, L., Human antibody responses to bovine (newbury-2) norovirus (GIII.2) and association to histo-blood group antigens. *J Med Virol* **2010,** 82, (7), 1241-1246.10.1002/jmv.21776
15. Mesquita, J. R.; Costantini, V. P.; Cannon, J. L.; Lin, S. C.; Nascimento, M. S. J.; Vinjé, J., Presence of Antibodies against Genogroup VI Norovirus in Humans. *Virol J* **2013,** 10.10.1186/1743-422x-10-176
16. Martino, B. D.; Di Profio, F.; Ceci, C.; Felice, E. D.; Green, K. Y.; Bok, K.; Grazia, S. D.; Giammanco, G. M.; Massirio, I.; Lorusso, E.; Buonavoglia, C.; Marsilio, F.; Martella, V., Seroprevalence of norovirus genogroup IV antibodies among humans, Italy, 2010-2011. *Emerg Infect Dis* **2014,** 20, (11), 1828-1832.10.3201/eid2011.131601
17. Smith, A. W.; Skilling, D. E.; Cherry, N.; Mead, J. H.; Matson, D. O., Calicivirus emergence from ocean reservoirs: Zoonotic and interspecies movements. *Emerg Infect Dis* **1998,** 4, (1), 13-20
18. Karst, S. M.; Wobus, C. E., Viruses in Rodent Colonies: Lessons Learned from Murine Noroviruses. *Annu Rev Virol* **2015,** 2, 525-548.10.1146/annurev-virology-100114-055204
19. Karst, S. M.; Wobus, C. E.; Goodfellow, I. G.; Green, K. Y.; Virgin, H. W., Advances in norovirus biology. *Cell Host and Microbe* **2014,** 15, (6), 668-680.10.1016/j.chom.2014.05.015
20. Baldridge, M. T.; Turula, H.; Wobus, C. E., Norovirus Regulation by Host and Microbe. *Trends Mol Med* **2016,** 22, (12), 1047-1059
21. Wang, J.; Deng, Z., Detection and forecasting of oyster norovirus outbreaks: Recent advances and future perspectives. *Mar Environ Res* **2012,** 80, 62-69
22. Hassard, F.; Harris, J. P.; Jones, D. L.; Sharp, J. H.; Taft, H.; LeVay, L.; McDonald, J. E.; Tuson, K.; Malham, S. K., Critical Review on the Public Health Impact of Norovirus Contamination in Shellfish and the Environment: A UK Perspective. **2017,** 9, 123–141
23. Adler, J. L.; Zickl, R., Winter Vomiting Disease *J Infect Dis* **1969,** 119, (6), 668-673
24. Kapikian, A. Z., The Discovery of the 27-nm Norwalk Virus: An Historic Perspective. *J Infect Dis* **2000,** 181 (Supplement_2), S295-S302
25. Wang, Q. H.; Myung, G. H.; Cheetham, S.; Souza, M.; Funk, J. A.; Saif, L. J., Porcine noroviruses related to human noroviruses. *Emerg Infect Dis* **2005,** 11, (12), 1874-1881.10.3201/eid1112.050485
26. Shen, Q.; Zhang, W.; Yang, S.; Cui, L.; Hua, X., Complete genome sequence of a new-genotype porcine norovirus isolated from piglets with diarrhea. *J Virol* **2012,** 86, (12), 7015-7016.10.1128/jvi.00757-12
27. Shen, Q.; Zhang, W.; Yang, S.; Yang, Z.; Chen, Y.; Cui, L.; Zhu, J.; Hua, X., Recombinant porcine norovirus identified from piglet with diarrhea. *BMC Vet Res* **2012,** 8, 155.10.1186/1746-6148-8-155
28. Scheuer, K. A.; Oka, T.; Hoet, A. E.; Gebreyes, W. A.; Molla, B. Z.; Saif, L. J.; Wang, Q., Prevalence of porcine Noroviruses, molecular characterization of emerging porcine sapoviruses from finisher swine in the United States, and unified classification scheme for sapoviruses. *J Clin Microbiol* **2013,** 51, (7), 2344-2353.10.1128/jcm.00865-13
29. Niendorf, S.; Klemm, U.; Marques, A. M.; Bock, C. T.; Höhne, M., Infection with the persistent murine norovirus strain MNV-S99 suppresses IFN-Beta release and activation of stat1 In vitro. *PLoS ONE* **2016,** 11, (6).10.1371/journal.pone.0156898
30. Mattison, K.; Shukla, A.; Cook, A.; Pollari, F.; Friendship, R.; Kelton, D.; Bidawid, S.; Farber, J. M., Human noroviruses in swine and cattle. *Emerg Infect Dis* **2007,** 13, (8), 1184-1188.10.3201/eid1308.070005

31. L'Homme, Y.; Sansregret, R.; Plante-Fortier, É.; Lamontagne, A. M.; Lacroix, G.; Ouardani, M.; Deschamps, J.; Simard, G.; Simard, C., Genetic diversity of porcine Norovirus and Sapovirus: Canada, 2005-2007. *Arch Virol* **2009,** 154, (4), 581-593.10.1007/s00705-009-0344-6
32. L'Homme, Y.; Sansregret, R.; Simard, C., Broad range RT-PCR assays targeting human noroviruses also detect swine noroviruses. *Food Microbiol* **2009,** 26, (5), 552-555.10.1016/j.fm.2009.03.011
33. Shen, Q.; Ren, R.; Zhang, W.; Yang, Z.; Yang, S.; Chen, Y.; Cui, L.; Hua, X., Prevalence of hepatitis E virus and porcine caliciviruses in pig farms of Guizhou province, China. *Hepat Mon* **2011,** 11, (6), 459-463PMC3212798
34. Chao, D. Y.; Wei, J. Y.; Chang, W. F.; Wang, J.; Wang, L. C., Detection of Multiple Genotypes of Calicivirus Infection in Asymptomatic Swine in Taiwan. *Zoonoses Public Health* **2012,** 59, (6), 434-444.10.1111/j.1863-2378.2012.01483.x
35. Shen, Q.; Zhang, W.; Yang, S.; Chen, Y.; Ning, H.; Shan, T.; Liu, J.; Yang, Z.; Cui, L.; Zhu, J.; Hua, X., Molecular detection and prevalence of porcine caliciviruses in eastern China from 2008 to 2009. *Arch Virol* **2009,** 154, (10), 1625-1630.10.1007/s00705-009-0487-5
36. Nakamura, K.; Saga, Y.; Iwai, M.; Obara, M.; Horimoto, E.; Hasegawa, S.; Kurata, T.; Okumura, H.; Nagoshi, M.; Takizawa, T., Frequent detection of noroviruses and sapoviruses in swine and high genetic diversity of porcine sapovirus in Japan during fiscal year 2008. *J Clin Microbiol* **2010,** 48, (4), 1215-1222.10.1128/jcm.02130-09
37. Song, Y. J.; Yu, J. N.; Nam, H. M.; Bak, H. R.; Lee, J. B.; Park, S. Y.; Song, C. S.; Seo, K. H.; Choi, I. S., Identification of genetic diversity of porcine Norovirus and Sapovirus in Korea. *Virus Genes* **2011,** 42, (3), 394-401.10.1007/s11262-011-0588-6
38. Wolf, S.; Williamson, W.; Hewitt, J.; Lin, S.; Rivera-Aban, M.; Ball, A.; Scholes, P.; Savill, M.; Greening, G. E., Molecular detection of norovirus in sheep and pigs in New Zealand farms. *Vet Microbiol* **2009,** 133, (1-2), 184-189.10.1016/j.vetmic.2008.06.019
39. Martínez, M. A.; Alcalá, A. C.; Carruyo, G.; Botero, L.; Liprandi, F.; Ludert, J. E., Molecular detection of porcine enteric caliciviruses in Venezuelan farms. *Vet Microbiol* **2006,** 116, (1-3), 77-84.10.1016/j.vetmic.2006.03.016
40. Collins, P. J.; Martella, V.; Buonavoglia, C.; O'Shea, H., Detection and characterization of porcine sapoviruses from asymptomatic animals in Irish farms. *Vet Microbiol* **2009,** 139, (1-2), 176-182.10.1016/j.vetmic.2009.05.013
41. Machnowska, P.; Ellerbroek, L.; Johne, R., Detection and characterization of potentially zoonotic viruses in faeces of pigs at slaughter in Germany. *Vet Microbiol* **2014,** 168, (1), 60-68.10.1016/j.vetmic.2013.10.018
42. Mijovski, J. Z.; Poljšak-Prijatelj, M.; Steyer, A.; Barlič-Maganja, D.; Koren, S., Detection and molecular characterisation of noroviruses and sapoviruses in asymptomatic swine and cattle in Slovenian farms. *Infec Genet Evol* **2010,** 10, (3), 413-420.10.1016/j.meegid.2009.11.010
43. Sisay, Z.; Djikeng, A.; Berhe, N.; Belay, G.; Abegaz, W. E.; Wang, Q. H.; Saif, L. J., First detection and molecular characterization of sapoviruses and noroviruses with zoonotic potential in swine in Ethiopia. *Arch Virol* **2016,** 161, (10), 2739-2747.10.1007/s00705-016-2974-9
44. Silva, P. F.; Alfieri, A. F.; Barry, A. F.; de Arruda Leme, R.; Gardinali, N. R.; van der Poel, W. H.; Alfieri, A. A., High frequency of porcine norovirus infection in finisher units of Brazilian pig-production systems. *Trop Anim Health Prod* **2015,** 47, (1), 237-241.10.1007/s11250-014-0685-3
45. Yin, Y.; Tohya, Y.; Ogawa, Y.; Numazawa, D.; Kato, K.; Akashi, H., Genetic analysis of calicivirus genomes detected in intestinal contents of piglets in Japan. *Arch Virol* **2006,** 151, (9), 1749-1759.10.1007/s00705-006-0750-y
46. Farkas, T.; Nakajima, S.; Sugieda, M.; Deng, X.; Zhong, W.; Jiang, X., Seroprevalence of noroviruses in swine. *J Clin Microbiol* **2005,** 43, (2), 657-661.10.1128/jcm.43.2.657-661.2005
47. Mesquita, J. R.; Delgado, I.; Costantini, V.; Heenemann, K.; Vahlenkamp, T. W.; Vinjé, J.; Nascimento, M. S. J., Seroprevalence of canine norovirus in 14 European countries. *Clin Vaccine Immunol* **2014,** 21, (6), 898-900.10.1128/cvi.00048-14
48. Sisay, Z.; Wang, Q.; Oka, T.; Saif, L., Prevalence and molecular characterization of porcine enteric caliciviruses and first detection of porcine kobuviruses in US swine. *Arch Virol* **2013,** 158, (7), 1583-1588.10.1007/s00705-013-1619-5

49. Thomas, C.; Jung, K.; Han, M. G.; Hoet, A.; Scheuer, K.; Wang, Q.; Saif, L. J., Retrospective serosurveillance of bovine norovirus (GIII.2) and nebovirus in cattle from selected feedlots and a veal calf farm in 1999 to 2001 in the United States. *Arch Virol* **2014,** 159, (1), 83-90.10.1007/s00705-013-1795-3
50. Cho, Y. I.; Han, J. I.; Wang, C.; Cooper, V.; Schwartz, K.; Engelken, T.; Yoon, K. J., Case-control study of microbiological etiology associated with calf diarrhea. *Vet Microbiol* **2013,** 166, (3-4), 375-385.10.1016/j.vetmic.2013.07.001
51. Wise, A. G.; Monroe, S. S.; Hanson, L. E.; Grooms, D. L.; Sockett, D.; Maes, R. K., Molecular characterization of noroviruses detected in diarrheic stools of Michigan and Wisconsin dairy calves: Circulation of two distinct subgroups. *Virus Res* **2004,** 100, (2), 165-177.10.1016/j.virusres.2003.11.014
52. Smiley, J. R.; Hoet, A. E.; Tråvén, M.; Tsunemitsu, H.; Saif, L. J., Reverse transcription-PCR assays for detection of bovine enteric caliciviruses (BEC) and analysis of the genetic relationships among BEC and human caliciviruses. *J Clin Microbiol* **2003,** 41, (7), 3089-3099.10.1128/jcm.41.7.3089-3099.2003
53. Pinto, P.; Wang, Q.; Chen, N.; Dubovi, E. J.; Daniels, J. B.; Millward, L. M.; Buonavoglia, C.; Martella, V.; Saif, L. J., Discovery and genomic characterization of noroviruses from a gastroenteritis outbreak in domestic cats in the us. *PLoS ONE* **2012,** 7, (2).10.1371/journal.pone.0032739
54. Zhang, W.; Li, L.; Deng, X.; Kapusinszky, B.; Pesavento, P. A.; Delwart, E., Faecal virome of cats in an animal shelter. *J Gen Virol* **2014,** 95, 2553-2564.10.1099/vir.0.069674-0
55. Li, L.; Shan, T.; Wang, C.; Côté, C.; Kolman, J.; Onions, D.; Gulland, F. M. D.; Delwart, E., The fecal viral flora of California sea lions. *J Virol* **2011,** 85, (19), 9909-9917.10.1128/jvi.05026-11
56. Alcalá, A. C.; Hidalgo, M. A.; Obando, C.; Vizzi, E.; Liprandi, F.; Ludert, J. E., Molecular identification of bovine enteric caliciviruses in Venezuela. *Acta Cient Venez* **2003,** 54, (2), 148-152
57. Ferragut, F.; Vega, C. G.; Mauroy, A.; Conceição-Neto, N.; Zeller, M.; Heylen, E.; Uriarte, E. L.; Bilbao, G.; Bok, M.; Matthijnssens, J.; Thiry, E.; Badaracco, A.; Parreño, V., Molecular detection of bovine Noroviruses in Argentinean dairy calves: Circulation of a tentative new genotype. *Infec Genet Evol* **2016,** 40, 144-150.10.1016/j.meegid.2016.02.034
58. Cunha, J. B.; de Mendonça, M. C. L.; Miagostovich, M. P.; Leite, J. P. G., First detection of porcine norovirus GII.18 in Latin America. *Res Vet Sci* **2010,** 89, (1), 126-129.10.1016/j.rvsc.2009.12.013
59. Cunha, J. B.; de Mendonça, M. C. L.; Miagostovich, M. P.; Leite, J. P. G., Genetic diversity of porcine enteric caliciviruses in pigs raised in Rio de Janeiro State, Brazil. *Arch Virol* **2010,** 155, (8), 1301-1305.10.1007/s00705-010-0695-z
60. das Merces Hernandez, J.; Stangarlin, D. C.; Siqueira, J. A. M.; de Souza Oliveira, D.; Portal, T. M.; Barry, A. F.; Dias, F. A.; de Matos, J. C. S.; Mascarenhas, J. D. P.; Gabbay, Y. B., Genetic diversity of porcine sapoviruses in pigs from the Amazon region of Brazil. *Arch Virol* **2014,** 159, (5), 927-933.10.1007/s00705-013-1904-3
61. Almeida, P. R.; Lorenzetti, E.; Cruz, R. S.; Watanabe, T. T.; Zlotowski, P.; Alfieri, A. A.; Driemeier, D., Diarrhea caused by rotavirus A, B, and C in suckling piglets from southern Brazil: molecular detection and histologic and immunohistochemical characterization. *J Vet Diagn Invest* **2018**, 1040638718756050.10.1177/1040638718756050
62. Castro, T. X.; Cubel Garcia, R. C. N.; Fumian, T. M.; Costa, E. M.; Mello, R.; White, P. A.; Leite, J. P. G., Detection and molecular characterization of caliciviruses (vesivirus and norovirus) in an outbreak of acute diarrhea in kittens from Brazil. *Vet J* **2015,** 206, (1), 115-117.10.1016/j.tvjl.2015.06.014
63. Guo, Z.; He, Q.; Yue, H.; Zhang, B.; Tang, C., First detection of Nebovirus and Norovirus from cattle in China. *Arch Virol* **2018,** 163, (2), 475-478.10.1007/s00705-017-3616-6
64. Yang, L.; Wang, Q.; Xu, L.; Tu, C.; Huang, X.; He, B., Detection and Characterization of a Novel Norovirus in Bats, China. *Virol Sin* **2018,** 33, (1), 100-103.10.1007/s12250-018-0010-9
65. Soma, T.; Nakagomi, O.; Nakagomi, T.; Mochizuki, M., Detection of norovirus and sapovirus from diarrheic dogs and cats in Japan. *Microbiol Immunol* **2015,** 59, (3), 123-128.10.1111/1348-0421.12223
66. Tsunesumi, N.; Sato, G.; Iwasa, M.; Kabeya, H.; Maruyama, S.; Tohya, Y., Novel murine norovirus-like genes in wild rodents in Japan. *J Vet Med Sci* **2012,** 74, (9), 1221-1224.10.1292/jvms.12-0106
67. Keum, H. O.; Moon, H. J.; Park, S. J.; Kim, H. K.; Rho, S. M.; Park, B. K., Porcine noroviruses and sapoviruses on Korean swine farms. *Arch Virol* **2009,** 154, (11), 1765-1774.10.1007/s00705-009-0501-y
68. Lyoo, K. S.; Jung, M. C.; Yoon, S. W.; Kim, H. K.; Jeong, D. G., Identification of canine norovirus in dogs in South Korea. *BMC Vet Res* **2018,** 14, (1), 413.10.1186/s12917-018-1723-6

69. Park, S. I.; Jeong, C.; Kim, H. H.; Park, S. H.; Park, S. J.; Hyun, B. H.; Yang, D. K.; Kim, S. K.; Kang, M. I.; Cho, K. O., Molecular epidemiology of bovine noroviruses in South Korea. *Vet Microbiol* **2007,** 124, (1-2), 125-133.10.1016/j.vetmic.2007.03.010
70. Pourasgari, F.; Kaplon, J.; Sanchooli, A.; Fremy, C.; Karimi-Naghlani, S.; Otarod, V.; Ambert-Balay, K.; Mojgani, N.; Pothier, P., Molecular prevalence of bovine noroviruses and neboviruses in newborn calves in Iran. **2018**.10.1007/s00705-018-3716-y
71. Mahzounieh, M.; Moghtadaei, E.; Zahraei Salehi, T., Detection of calicivirus genomes in calves using Ni/E3 primers in Shahrekord Area, Iran. *Pakistan Journal of Biological Sciences* **2006,** 9, (2), 227-230
72. Yilmaz, H.; Turan, N.; Altan, E.; Bostan, K.; Yilmaz, A.; Helps, C. R.; Cho, K. O., First report on the phylogeny of bovine norovirus in Turkey. *Arch Virol* **2011,** 156, (1), 143-147.10.1007/s00705-010-0833-7
73. Thuran, T.; Irehan, B., Detection and molecular analysis of bovine enteric norovirus and nebovirus in Turkey. *J Vet Res* **2018,** 62, (2), 129-135.10.2478/jvetres-2018-0021
74. Haga, K.; Fujimoto, A.; Takai-Todaka, R.; Miki, M.; Doan, Y. H.; Murakami, K.; Yokoyama, M.; Murata, K.; Nakanishi, A.; Katayama, K., Functional receptor molecules CD300lf and CD300ld within the CD300 family enable murine noroviruses to infect cells. *Proc Natl Acad Sci U S A* **2016,** 113, (41), E6248-E6255.10.1073/pnas.1605575113
75. Wolf, S.; Williamson, W. M.; Hewitt, J.; Rivera-Aban, M.; Lin, S.; Ball, A.; Scholes, P.; Greening, G. E., Sensitive multiplex real-time reverse transcription-PCR assay for the detection of human and animal noroviruses in clinical and environmental samples. *Appl Environ Microbiol* **2007,** 73, (17), 5464-5470.10.1128/aem.00572-07
76. Monini, M.; Di Bartolo, I.; Ianiro, G.; Angeloni, G.; Magistrali, C. F.; Ostanello, F.; Ruggeri, F. M., Detection and molecular characterization of zoonotic viruses in swine fecal samples in Italian pig herds. *Arch Virol* **2015,** 160, (10), 2547-2556.10.1007/s00705-015-2538-4
77. Di Bartolo, I.; Tofani, S.; Angeloni, G.; Ponterio, E.; Ostanello, F.; Ruggeri, F. M., Detection and characterization of porcine caliciviruses in Italy. *Arch Virol* **2014,** 159, (9), 2479-2484.10.1007/s00705-014-2076-5
78. Di Martino, B.; Di Profio, F.; Di Felice, E.; Melegari, I.; Ceci, C.; Mauroy, A.; Thiry, E.; Martella, V.; Marsilio, F., Genetic heterogeneity of bovine noroviruses in Italy. *Arch Virol* **2014,** 159, (10), 2717-2722.10.1007/s00705-014-2109-0
79. Di Bartolo, I.; Ponterio, E.; Monini, M.; Ruggeri, F. M., A pilot survey of bovine norovirus in northern Italy. *Vet Rec* **2011,** 169, (3), 73.10.1136/vr.d2625
80. Di Martino, B.; Di Profio, F.; Melegari, I.; Sarchese, V.; Massirio, I.; Palermo, G.; Romito, G.; Lorusso, E.; Lanave, G.; Bodnar, L.; Buonavoglia, C.; Marsilio, F.; Green, K. Y.; Martella, V., Seroprevalence for norovirus genogroup II, IV and VI in dogs. *Vet Microbiol* **2017,** 203, 68-72.10.1016/j.vetmic.2017.03.006
81. Di Martino, B.; Marsilio, F.; Di Profio, F.; Lorusso, E.; Friedrich, K. G.; Buonavoglia, C.; Martella, V., Detection of antibodies against Norovirus genogroup GIV in carnivores. *Clin Vaccine Immunol* **2010,** 17, (1), 180-182.10.1128/cvi.00312-09
82. Martella, V.; Decaro, N.; Lorusso, E.; Radogna, A.; Moschidou, P.; Amorisco, F.; Lucente, M. S.; Desario, C.; Mari, V.; Elia, G.; Banyai, K.; Carmichael, L. E.; Buonavoglia, C., Genetic heterogeneity and recombination in canine noroviruses. *J Virol* **2009,** 83, (21), 11391-11396.10.1128/jvi.01385-09
83. Bodnar, L.; Lorusso, E.; Di Martino, B.; Catella, C.; Lanave, G.; Elia, G.; Bányai, K.; Buonavoglia, C.; Martella, V., Identification of a novel canine norovirus. *Infec Genet Evol* **2017,** 52, 75-81.10.1016/j.meegid.2017.04.020
84. Martella, V.; Campolo, M.; Lorusso, E.; Cavicchio, P.; Camero, M.; Bellacicco, A. L.; Decaro, N.; Elia, G.; Greco, G.; Corrente, M.; Desario, C.; Arista, S.; Banyai, K.; Koopmans, M.; Buonavoglia, C., Norovirus in captive lion cub (Panthera leo). *Emerg Infect Dis* **2007,** 13, (7), 1071-1073.10.3201/eid1307.070268.
85. Di Martino, B.; Di Profio, F.; Melegari, I.; Sarchese, V.; Cafiero, M. A.; Robetto, S.; Aste, G.; Lanave, G.; Marsilio, F.; Martella, V., A novel feline norovirus in diarrheic cats. *Infec Genet Evol* **2016,** 38, 132-137.10.1016/j.meegid.2015.12.019
86. Halaihel, N.; Masía, R. M.; Fernández-Jiménez, M.; Ribes, J. M.; Montava, R.; De Blas, I.; Gironés, O.; Alonso, J. L.; Buesa, J., Enteric calicivirus and rotavirus infections in domestic pigs. *Epidemiol Infect* **2010,** 138, (4), 542-548.10.1017/s0950268809990872
87. Mesquita, J. R.; Nascimento, M. S., Molecular epidemiology of canine norovirus in dogs from Portugal, 2007-2011. *BMC Vet Res* **2012,** 8, 107.10.1186/1746-6148-8-107.

88. Mesquita, J. R.; Nascimento, M. S. J., Gastroenteritis outbreak associated with faecal shedding of canine norovirus in a portuguese kennel following introduction of imported dogs from russia. *Transboundary Emer Dis* **2012,** 59, (5), 456-459.10.1111/j.1865-1682.2011.01284.x
89. Mesquita, J. R.; Barclay, L.; Nascimento, M. S. J.; Vinjé, J., Novel norovirus in dogs with diarrhea. *Emerg Infect Dis* **2010,** 16, (6), 980-982.10.3201/eid1606.091861
90. Ntafis, V.; Xylouri, E.; Radogna, A.; Buonavoglia, C.; Martella, V., Outbreak of canine norovirus infection in young dogs. *J Clin Microbiol* **2010,** 48, (7), 2605-2608.10.1128/jcm.02528-09
91. Kaplon, J.; Guenau, E.; Asdrubal, P.; Pothier, P.; Ambert-Balay, K., Possible novel Nebovirus genotype in cattle, France. *Emerg Infect Dis* **2011,** 17, (6), 1120-1123.10.3201/eid1706.100038
92. Kaplon, J.; Fremy, C.; Bernard, S.; Rehby, L.; Aho, S.; Pothier, P.; Ambert-Balay, K., Impact of rotavirus vaccine on rotavirus genotypes and caliciviruses circulating in French cattle. *Vaccine* **2013,** 31, (20), 2433-2440.10.1016/j.vaccine.2013.03.039
93. Oliver, S. L.; Wood, E.; Asobayire, E.; Wathes, D. C.; Brickell, J. S.; Elschner, M.; Otto, P.; Lambden, P. R.; Clarke, I. N.; Bridger, J. C., Serotype 1 and 2 bovine noroviruses are endemic in cattle in the United Kingdom and Germany. *J Clin Microbiol* **2007,** 45, (9), 3050-3052.10.1128/jcm.02015-06
94. Deng, Y.; Batten, C. A.; Liu, B. L.; Lambden, P. R.; Elschner, M.; Günther, H.; Otto, H. P.; Schnürch, P.; Eichhorn, W.; Herbst, W.; Clarke, I. N., Studies of epidemiology and seroprevalence of bovine Noroviruses in Germany. *J Clin Microbiol* **2003,** 41, (6), 2300-2305.10.1128/JCM.41.6.2300–2305.200
95. Ike, A. C.; Roth, B. N.; Böhm, R.; Pfitzner, A. J.; Marschang, R. E., Identification of bovine enteric Caliciviruses (BEC) from cattle in Baden-Württemberg. *DTW Dtsch Tierarztl Wochenschr* **2007,** 114, (1), 12-15
96. Sachsenröder, J.; Braun, A.; Machnowska, P.; Ng, T. F. F.; Deng, X.; Guenther, S.; Bernstein, S.; Ulrich, R. G.; Delwart, E.; Johne, R., Metagenomic identification of novel enteric viruses in urban wild rats and genome characterization of a group A rotavirus. *J Gen Virol* **2014,** 95, 2734-2747.10.1099/vir.0.070029-0
97. Van Der Poel, W. H. M.; Van Der Heide, R.; Verschoor, F.; Gelderblom, H.; Vinjé, J.; Koopmans, M. P. G., Epidemiology of Norwalk-like virus infections in cattle in The Netherlands. *Vet Microbiol* **2003,** 92, (4), 297-309.10.1016/s0378-1135(02)00421-2
98. de Graaf, M.; Bodewes, R.; van Elk, C. E.; van de Bildt, M.; Getu, S.; Aron, G. I.; Verjans, G. M. G. M.; Osterhaus, A. D. M. E.; van den Brand, J. M. A.; Kuiken, T.; Koopmans, M. P. G., Norovirus infection in harbor porpoises. *Emerg Infect Dis* **2017,** 23, (1), 87-91.10.3201/eid2301.161081
99. Mauroy, A.; Scipioni, A.; Mathijs, E.; Miry, C.; Ziant, D.; Thys, C.; Thiry, E., Noroviruses and sapoviruses in pigs in Belgium. *Arch Virol* **2008,** 153, (10), 1927-1931.10.1007/s00705-008-0189-4
100. Mauroy, A.; Scipioni, A.; Mathijs, E.; Saegerman, C.; Mast, J.; Bridger, J. C.; Ziant, D.; Thys, C.; Thiry, E., Epidemiological study of bovine norovirus infection by RT-PCR and a VLP-based antibody ELISA. *Vet Microbiol* **2009,** 137, (3-4), 243-251.10.1016/j.vetmic.2009.01.031
101. Mauroy, A.; Scipioni, A.; Mathijs, E.; Ziant, D.; Daube, G.; Thirya, E., Complete genome sequence of a novel bovine norovirus: Evidence for slow genetic evolution in genogroup III genotype 2 noroviruses. *J Virol* **2012,** 86, (22), 12449-12450.10.1128/jvi.02251-12
102. Mauroy, A.; Scipioni, A.; Mathijs, E.; Thys, C.; Thiry, E., Molecular detection of kobuviruses and recombinant noroviruses in cattle in continental Europe. *Arch Virol* **2009,** 154, (11), 1841-1845.10.1007/s00705-009-0518-2
103. Woode, G. N.; Bridger, J. C., Isolation of small viruses resembling astroviruses and caliciviruses from acute enteritis of calves. *J Med Microbiol* **1978,** 11, (4), 441-452
104. Milnes, A. S.; Binns, S. H.; Oliver, S. L.; Bridger, J. C., Retrospective study of noroviruses in samples of diarrhoea from cattle, using the Veterinary Laboratories Agency's Farmfile database. *Vet Rec* **2007,** 160, (10), 326-330.10;160(10):326-30.
105. Caddy, S.; Emmott, E.; El-Attar, L.; Mitchell, J.; De Rougemont, A.; Brownlie, J.; Goodfellow, I., Serological evidence for multiple strains of canine norovirus in the UK dog population. *PLoS ONE* **2013,** 8, (12).10.1371/journal.pone.0081596
106. Caddy, S. L.; De Rougemont, A.; Emmott, E.; El-Attar, L.; Mitchell, J. A.; Hollinshead, M.; Belliot, G.; Brownlie, J.; Le Pendu, J.; Goodfellow, I., Evidence for human norovirus infection of dogs in the United Kingdom. *J Clin Microbiol* **2015,** 53, (6), 1873-1883.10.1128/jcm.02778-14

107. Smith, D. B.; McFadden, N.; Blundell, R. J.; Meredith, A.; Simmonds, P., Diversity of murine norovirus in wild-rodent populations: Species-specific associations suggest an ancient divergence. *J Gen Virol* **2012,** 93, (2), X259-266.10.1099/vir.0.036392-0
108. Wolf, S.; Reetz, J.; Johne, R.; Heiberg, A. C.; Petri, S.; Kanig, H.; Ulrich, R. G., The simultaneous occurrence of human norovirus and hepatitis E virus in a Norway rat (Rattus norvegicus). *Arch Virol* **2013,** 158, (7), 1575-1578.10.1007/s00705-013-1646-2
109. Jor, E.; Myrmel, M.; Jonassen, C. M., SYBR Green based real-time RT-PCR assay for detection and genotype prediction of bovine noroviruses and assessment of clinical significance in Norway. *J Virol Methods* **2010,** 169, (1), 1-7.10.1016/j.jviromet.2010.03.028
110. Summa, M.; von Bonsdorff, C. H.; Maunula, L., Pet dogs-A transmission route for human noroviruses? *J Clin Virol* **2012,** 53, (3), 244-247.10.1016/j.jcv.2011.12.014
111. Summa, M.; Henttonen, H.; Maunula, L., Human noroviruses in the faeces of wild birds and rodents-new potential transmission routes. *Zoonoses Public Health* **2018**.10.1111/zph.12461
112. Reuter, G.; Bíró, H.; Szucs, G., Enteric caliciviruses in domestic pigs in Hungary. *Arch Virol* **2007,** 152, (3), 611-614.10.1007/s00705-006-0887-8
113. Mihalov-Kovács, E.; Marton, S.; Fehér, E.; Lengyel, G.; Jakab, F.; Tuboly, T.; Bányai, K., Enteric viral infections of sheltered dogs in Hungary. *Magyar Allatorvosok Lapja* **2014,** 136, 661-670
114. Farkas, T.; Fey, B.; Keller, G.; Martella, V.; Egyed, L., Molecular detection of murine noroviruses in laboratory and wild mice. *Vet Microbiol* **2012,** 160, (3-4), 463-467.10.1016/j.vetmic.2012.06.002
115. Mohamed, F. F.; Mansour, S. M.; El-Araby, I. E.; Mor, S. K.; Goyal, S. M., Molecular detection of enteric viruses from diarrheic calves in Egypt. *Arch Virol* **2017,** 162, (1), 129-137.10.1007/s00705-016-3088-0
116. Hassine-Zaafrane, M.; Kaplon, J.; Sdiri-Loulizi, K.; Aouni, Z.; Pothier, P.; Aouni, M.; Ambert-Balay, K., Molecular prevalence of bovine noroviruses and neboviruses detected in central-eastern Tunisia. *Arch Virol* **2012,** 157, (8), 1599-1604.10.1007/s00705-012-1344-5
117. Taku, O.; Iweriebor, B. C.; Nwodo, U. U.; Obi, L. C.; Okoh, A. I., Occurrence of Norovirus in pig faecal samples in the Eastern Cape, South Africa. *Asian Pac J Trop Dis* **2017,** 7, (3), 151-155.10.12980/apjtd.7.2017D6-393
118. Günther, H.; Otto, P.; Heilmann, P., Diarrhea in young calves. 6. Determination of the pathogenicity of a bovine coronavirus and an unidentified icosahedral virus. *Arch Exp Veterinarmed* **1984,** 38, (5), 781-792
119. Jung, K.; Scheuer, K. A.; Zhang, Z.; Wang, Q.; Saif, L. J., Pathogenesis of GIII.2 bovine norovirus, CV186-OH/00/US strain in gnotobiotic calves. *Vet Microbiol* **2014,** 168, (1), 202-207.10.1016/j.vetmic.2013.11.008
120. Otto, P. H.; Clarke, I. N.; Lambden, P. R.; Salim, O.; Reetz, J.; Liebler-Tenorio, E. M., Infection of calves with bovine norovirus GIII.1 strain Jena virus: An experimental model to study the pathogenesis of norovirus infection. *J Virol* **2011,** 85, (22), 12013-12021.10.1128/jvi.05342-11
121. Han, M. G.; Wang, Q.; Smiley, J. R.; Change, K. O.; Saif, L. J., Self-assembly of the recombinant capsid protein of a bovine norovirus (BoNV) into virus-like particles and evaluation of cross-reactivity of BoNV with human noroviruses. *J Clin Microbiol* **2005,** 43, (2), 778-785.10.1128/jcm.43.2.778-785.2005
122. Han, M. G.; Cheetham, S.; Azevedo, M.; Thomas, C.; Saif, L. J., Immune responses to bovine norovirus-like particles with various adjuvants and analysis of protection in gnotobiotic calves. *Vaccine* **2006,** 24, (3), 317-326.10.1016/j.vaccine.2005.07.071
123. Souza, M.; Azevedo, M. S. P.; Jung, K.; Cheetham, S.; Saif, L. J., Pathogenesis and immune responses in gnotobiotic calves after infection with the genogroup II.4-HS66 strain of human norovirus. *J Virol* **2008,** 82, (4), 1777-1786.10.1128/jvi.01347-07
124. Oliver, S. L.; Asobayire, E.; Charpilienne, A.; Cohen, J.; Bridger, J. C., Complete genomic characterization and antigenic relatedness of genogroup III, genotype 2 bovine noroviruses. *Arch Virol* **2007,** 152, (2), 257-272.10.1007/s00705-006-0856-2
125. La Rosa, G.; Iaconelli, M.; Pourshaban, M.; Fratini, M., Molecular detection and genetic diversity of norovirus genogroup IV: a yearlong monitoring of sewage throughout Italy. *Arch Virol* **2010,** 155, (4), 589-593.10.1007/s00705-010-0619-y
126. van Beek, J.; de Graaf, M.; Al-Hello, H.; Allen, D. J.; Ambert-Balay, K.; Botteldoorn, N.; Brytting, M.; Buesa, J.; Cabrerizo, M.; Chan, M.; Cloak, F.; Di Bartolo, I.; Guix, S.; Hewitt, J.; Iritani, N.; Jin, M.; Johne, R.; Lederer, I.; Mans, J.; Martella, V.; Maunula, L.; McAllister, G.; Niendorf, S.; Niesters, H. G.; Podkolzin, A. T.; Poljsak-Prijatelj, M.; Rasmussen, L. D.; Reuter, G.; Tuite, G.; Kroneman, A.; Vennema, H.; Koopmans, M. P. G.;

NoroNet, Molecular surveillance of norovirus, 2005–16: an epidemiological analysis of data collected from the NoroNet network. *Lancet Infect Dis* **2018,** 18, (5), 545-553
127. Takano, T.; Kusuhara, H.; Kuroishi, A.; Takashina, M.; Doki, T.; Nishinaka, T.; Hohdatsu, T., Molecular characterization and pathogenicity of a genogroup GVI feline norovirus. *Vet Microbiol* **2015,** 178, (3-4), 201-207.10.1016/j.vetmic.2015.05.018
128. Takano, T.; Hiramatsu, K.; Matsuyama, M.; Mutoh, K.; Matsumoto, Y.; Fukushima, T.; Doki, T.; Kusuhara, H.; Hohdatsu, T., Viral shedding and clinical status of feline-norovirus-infected cats after reinfection with the same strain. *Arch Virol* **2018,** 163, (6), 1503-1510.10.1007/s00705-018-3770-5
129. Kocher, J. F.; Lindesmith, L. C.; Huynh, J.; Gates, J. E.; Debbink, K.; Beall, A.; Mallory, M. L.; Donaldson, E. F.; Barica, R. S., Bat Caliciviruses and Human Noroviruses Are Antigenically Similar and Have Overlapping Histo-Blood Group Antigen Binding Profile. *MBio* **2018,** 9, (3), e00869-18
130. Wu, Z.; Yang, L.; Ren, X.; He, G.; Zhang, J.; Yang, J.; Qian, Z.; Dong, J.; Sun, L.; Zhu, Y.; Du, J.; Yang, F.; Zhang, S.; Jin, Q., Deciphering the bat virome catalog to better understand the ecological diversity of bat viruses and the bat origin of emerging infectious diseases. *ISME J* **2016,** 10, (3), 609-620.10.1038/ismej.2015.138
131. Humphrey, T. J., An outbreak of calicivirus associated gastroenteritis in an elderly persons home. A possible zoonosis? *J Hyg (Lond)* **1984,** 93, (2), 293-299PMC2129430
132. Widdowson, M. A.; Rockx, B.; Schepp, R.; Van Der Poel, W. H. M.; Vinje, J.; Van Duynhoven, Y. T.; Koopmans, M. P., Detection of serum antibodies to bovine norovirus in veterinarians and the general population in The Netherlands. *J Med Virol* **2005,** 76, (1), 119-128.10.1002/jmv.20333
133. Bucardo, F.; González, F.; Reyes, Y.; Blandón, P.; Saif, L.; Nordgren, J., Seroprevalence in Household Raised Pigs Indicate High Exposure to GII Noroviruses in Rural Nicaragua. *Zoonoses Public Health* **2016,** 63, (8), 600-607.10.1111/zph.12269
134. Chan, M. C. W.; Hu, Y.; Chen, H.; Podkolzin, A. T.; Zaytseva, E. V.; Komano, J.; Sakon, N.; Poovorawan, Y.; Vongpunsawad, S.; Thanusuwannasak, T.; Hewitt, J.; Croucher, D.; Collins, N.; Vinjé, J.; Pang, X. L.; Lee, B. E.; de Graaf, M.; van Beek, J.; Vennema, H.; Koopmans, M. P. G.; Niendorf, S.; Poljsak-Prijatelj, M.; Steyer, A.; White, P. A.; Lun, J. H.; Mans, J.; Hung, T. N.; Kwok, K.; Cheung, K.; Lee, N.; Chan, P. K. S., Global Spread of Norovirus GII.17 Kawasaki 308, 2014–2016. *Emerg Infect Dis* **2017,** 23, (8), 1350-1354.10.3201/eid2308.161138.
135. de Graaf, M.; van Beek, J.; Vennema, H.; Podkolzin, A. T.; Hewitt, J.; Bucardo, F.; Templeton, K.; Nordgren, J.; Reuter, G.; Lynch, M.; Rasmussen, L. D.; Iritani, N.; Chan, M. C.; Martella, V.; Ambert-Balay, K.; Vinjé, J.; White, P. A.; Koopmans, M. P., Emergence of a novel GII.17 norovirus - End of the GII.4 era? *Euro Surveill* **2015,** 20, (26), 1-8PMC5921880
136. He, Z.; Liu, B.; Tao, Y.; Li, C.; Xia, M.; Zhong, W.; Jiang, X.; Liu, H.; Tan, M., Norovirus GII.17 natural infections in Rhesus monkeys, China. *Emerg Infect Dis* **2017,** 23, (2), 316-319.10.3201/eid2302.161077
137. Liu, B.; Tao, Y.; Li, C.; Li, X.; Liu, J.; He, Z.; Xia, M.; Jiang, X.; Tan, M.; Liu, H., Complete Genome Sequence of a GII.17 Norovirus Isolated from a Rhesus Monkey in China. *Genome Announc* **2016,** 4, (5).10.1128/genomeA.00904-16.
138. Farkas, T.; Cross, R. W.; Hargitt, I. E.; Lerche, N. W.; Morrow, A. L.; Sestak, K., Genetic diversity and histo-blood group antigen interactions of rhesus enteric caliciviruses. *J Virol* **2010,** 84, (17), 8617-8625.10.1128/jvi.00630-10
139. Farkas, T., Natural norovirus infections in rhesus macaques. *Emerg Infect Dis* **2016,** 22, (7), 1272-1274.10.3201/eid2207.151740
140. Jiang, B.; McClure, H. M.; Fankhauser, R. L.; Monroe, S. S.; Glass, R. I., Prevalence of rotavirus and norovirus antibodies in non-human primates. *J Med Primatol* **2004,** 33, (1), 30-33.10.1111/j.1600-0684.2003.00051.x
141. Farkas, T.; Dofour, J.; Jiang, X.; Sestak, K., Detection of norovirus-, sapovirus- and rhesus enteric calicivirus-specific antibodies in captive juvenile macaques. *J Gen Virol* **2010,** 91, (3), 734-738.10.1099/vir.0.015263-0
142. Rockx, B. H. G.; Bogers, W. M. J. M.; Heeney, J. L.; Van Amerongen, G.; Koopmans, M. P. G., Experimental norovirus infections in non-human primates. *J Med Virol* **2005,** 75, (2), 313-320.10.1002/jmv.20273
143. de Graaf, M.; van Beek, J.; Koopmans, M. P. G., Human norovirus transmission and evolution in a changing world. *Nat Rev Microbiol* **2016,** 14, (7), 421-433.10.1038/nrmicro.2016.48

144. Taube, S.; Kolawole, A. O.; Höhne, M.; Wilkinson, J. E.; Handley, S. A.; Perry, J. W.; Thackray, L. B.; Akkina, R.; Wobus, C. E., A mouse model for human norovirus. *mBio* **2013,** 4, (4).10.1128/mBio.00450-13
145. Seo, D. J.; Jung, D.; Jung, S.; Ha, S. K.; Ha, S. D.; Choi, I. S.; Myoung, J.; Choi, C., Experimental miniature piglet model for the infection of human norovirus GII. *J Med Virol* **2018,** 90, (4), 655-662.10.1002/jmv.24991
146. Cheetham, S.; Souza, M.; McGregor, R.; Meulia, T.; Wang, Q.; Saif, L. J., Binding patterns of human norovirus-like particles to buccal and intestinal tissues of gnotobiotic pigs in relation to A/H histo-blood group antigen expression. *J Virol* **2007,** 81, (7), 3535-3544.10.1128/jvi.01306-06
147. Jones, M. K.; Watanabe, M.; Zhu, S.; Graves, C. L.; Keyes, L. R.; Grau, K. R.; Gonzalez-Hernandez, M. B.; Iovine, N. M.; Wobus, C. E.; Vinjé, J.; Tibbetts, S. A.; Wallet, S. M.; Karst, S. M., Enteric bacteria promote human and mouse norovirus infection of B cells. *Science* **2014,** 346, (6210), 755-759.10.1126/science.1257147
148. Ettayebi, K.; Crawford, S. E.; Murakami, K.; Broughman, J. R.; Karandikar, U.; Tenge, V. R.; Neill, F. H.; Blutt, S. E.; Zeng, X. L.; Qu, L.; Kou, B.; Opekun, A. R.; Burrin, D.; Graham, D. Y.; Ramani, S.; Atmar, R. L.; Estes, M. K., Replication of human noroviruses in stem cell–derived human enteroids. *SCIENCE* **2016,** 353, (6306), 1387-1392
149. Duizer, E.; Schwab, K. J.; Neill, F. H.; Atmar, R. L.; Koopmans, M. P. G.; Estes, M. K., Laboratory efforts to cultivate noroviruses. *J Gen Virol* **2004,** 85, (1), 79-87.10.1099/vir.0.19478-0
150. Oka, T.; Stoltzfus, G. T.; Zhu, C.; Jung, K.; Wang, Q.; Saif, L. J., Attempts to grow human noroviruses, a sapovirus, and a bovine norovirus in vitro. *PLoS ONE* **2018,** 13, (2).10.1371/journal.pone.0178157
151. Lou, F.; Ye, M.; Ma, Y.; Li, X.; DiCaprio, E.; Chen, H.; Krakowka, S.; Hughes, J.; Kingsley, D.; Li, J., A Gnotobiotic Pig Model for Determining Human Norovirus Inactivation by High-Pressure Processing. *Appl Environ Microbiol* **2015,** 81, (19), 6679-6687.10.1128/aem.01566-15
152. Bui, T.; Kocher, J.; Li, Y.; Wen, K.; Li, G.; Liu, F.; Yang, X.; Leroith, T.; Tan, M.; Xia, M.; Zhong, W.; Jiang, X.; Yuan, L., Median infectious dose of human norovirus GII.4 in gnotobiotic pigs is decreased by simvastatin treatment and increased by age. *J Gen Virol* **2013,** 94, (PART9), 2005-2016.10.1099/vir.0.054080-0
153. Kocher, J.; Bui, T.; Giri-Rachman, E.; Wen, K.; Li, G.; Yang, X.; Liu, F.; Tan, M.; Xia, M.; Zhong, W.; Jiang, X.; Yuan, L., Intranasal P particle vaccine provided partial cross-variant protection against human GII.4 Norovirus diarrhea in gnotobiotic pigs. *J Virol* **2014,** 8, (17), 9728-9743.10.1128/jvi.01249-14
154. Lei, S.; Ramesh, A.; Twitchell, E.; Wen, K.; Bui, T.; Weiss, M.; Yang, X.; Kocher, J.; Li, G.; Giri-Rachman, E.; Van Trang, N.; Jiang, X.; Ryan, E. P.; Yuan, L., High protective efficacy of probiotics and rice bran against human norovirus infection and diarrhea in gnotobiotic pigs. *Front Microbiol* **2016,** 7, (NOV).10.3389/fmicb.2016.01699
155. Lei, S.; Ryu, J.; Wen, K.; Twitchell, E.; Bui, T.; Ramesh, A.; Weiss, M.; Li, G.; Samuel, H.; Clark-Deener, S.; Jiang, X.; Lee, K.; Yuan, L., Increased and prolonged human norovirus infection in RAG2/IL2RG deficient gnotobiotic pigs with severe combined immunodeficiency. *Sci Rep* **2016,** 6, 25222.10.1038/srep25222
156. Takanashi, S.; Wang, Q.; Chen, N.; Shen, Q.; Jung, K.; Zhang, Z.; Yokoyama, M.; Lindesmith, L. C.; Baric, R. S.; Saif, L. J., Characterization of emerging GII.g/GII.12 noroviruses from a gastroenteritis outbreak in the United States in 2010. *J Clin Microbiol* **2011,** 49, (9), 3234-3244.10.1128/jcm.00305-11
157. Souza, M.; Cheetham, S. M.; Azevedo, M. S. P.; Costantini, V.; Saif, L. J., Cytokine and antibody responses in gnotobiotic pigs after infection with human norovirus genogroup II.4 (HS66 strain). *J Virol* **2007,** 81, (17), 9183-9192.10.1128/jvi.00558-07
158. Lei, S.; Samuel, H.; Twitchell, E.; Bui, T.; Ramesh, A.; Wen, K.; Weiss, M.; Li, G.; Yang, X.; Jiang, X.; Yuan, L., Enterobacter cloacae inhibits human norovirus infectivity in gnotobiotic pigs. *Sci Rep* **2016,** 6, 25017.10.1038/srep25017
159. Cheetham, S.; Menira, S.; Meulia, T.; Grimes, S.; Han, M. G.; Saif, L. J., Pathogenesis of a genogroup II human norovirus in gnotobiotic pigs. *J Virol* **2006,** 80, (21), 10372-81.10.1128/JVI.00809-06
160. Park, B. J.; Jung, S. T.; Choi, C. S.; Myoung, J.; Ahn, H. S.; Han, S. H.; Kim, Y. H.; Go, H. J.; Lee, J. B.; Park, S. Y.; Song, C. S.; Lee, S. W.; Choi, I. S., Pathogenesis of Human Norovirus Genogroup II Genotype 4 in PostWeaning Gnotobiotic Pigs. *J Microbiol Biotechnol* **2018,** 28, (12), 2133–2140
161. Sestak, K.; Feely, S.; Fey, B.; Dufour, J.; Hargitt, E.; Alvarez, X.; Pahar, B.; Gregoricus, N.; Vinjé, J.; Farkas, T., Experimental inoculation of juvenile rhesus macaques with primate enteric caliciviruses. *PLoS ONE* **2012,** 7, (5).10.1371/journal.pone.0037973
162. Subekti, D. S.; Tjaniadi, P.; Lesmana, M.; McArdle, J.; Iskandriati, D.; Budiarsa, I. N.; Walujo, P.; Suparto, I. H.; Winoto, I.; Campbell, J. R.; Porter, K. R.; Sajuthi, D.; Ansari, A. A.; Oyofo, B. A., Experimental infection

of Macaca nemestrina with a Toronto Norwalk-like virus of epidemic viral gastroenteritis. *J Med Virol* **2002,** 66, (3), 400-406.10.1002/jmv.2159
163. Bok, K.; Parra, G. I.; Mitra, T.; Abente, E.; Shaver, C. K.; Boon, D.; Engle, R.; Yu, C.; Kapikian, A. Z.; Sosnovtsev, S. V.; Purcell, R. H.; Green, K. Y., Chimpanzees as an animal model for human norovirus infection and vaccine development. *Proc Natl Acad Sci U S A* **2011,** 108, (1), 325-330.10.1073/pnas.1014577107
164. Wyatt, R. G.; Greenberg, H. B.; Dalgard, D. W.; Allen, W. P.; Sly, D. L.; Thornhill, T. S.; Chanock, R. M.; Kapikian, A. Z., Experimental infection of chimpanzees with the Norwalk agent of epidemic viral gastroenteritis. *J Med Virol* **1978,** 2, (2), 89-96
165. Marionneau, S.; Cailleau-Thomas, A.; Rocher, J.; Le Moullac-Vaidye, B.; Ruvoën, N.; Monique, C.; Le Pendu, J., ABH and Lewis histo-blood group antigens, a model for the meaning of oligosaccharide diversity in the face of a changing world. *Biochimie* **2004,** 83, (7), 565-573
166. Huang, P.; Farkas, T.; Marionneau, S.; Zhong, W.; Ruvoën-Clouet, N.; Morrow, A. L.; Altaye, M.; Pickering, L. K.; Newburg, D. S.; LePendu, J.; Jiang, X., Noroviruses Bind to Human ABO, Lewis, and Secretor Histo–Blood Group Antigens: Identification of 4 Distinct Strain-Specific Patterns. *J Infect Dis* **2002,** 188, (1), 19-31.10.1086/375742
167. Ming, T.; Jiang, X., Histo-blood group antigens: a common niche for norovirus and rotavirus. *Expert Rev Mol Med* **2014,** 16,
168. Tan, M.; Jiang, X., Norovirus Gastroenteritis, Carbohydrate Receptors, and Animal Models. *PLoS Pathog* **2010,** 6, (6).10.1371/journal.ppat.1000983
169. Huang, P.; Farkas, T.; Zhong, W.; Tan, M.; Thornton, S.; Morrow, A. L.; Jiang, X., Norovirus and Histo-Blood Group Antigens: Demonstration of a Wide Spectrum of Strain Specificities and Classification of Two Major Binding Groups among Multiple Binding Patterns. *J Virol* **2005,** 79, (11), 6714-6722.10.1128/JVI.79.11.6714-6722.2005
170. Marionneau, S.; Ruvoën, N.; Le Moullac-Vaidye, B.; Clement, M.; Cailleau-Thomas, A.; Ruiz-Palacois, G.; Huang, P.; Jiang, X.; Le Pendu, J., Norwalk Virus Binds to Histo-Blood Group Antigens Present on Gastroduodenal Epithelial Cells of Secretor Individuals. *Gastroenterology* **2002,** 122, (7), 1967–1977
171. Tamura, M.; Natori, K.; Kobayashi, M.; Miyamura, T.; Takeda, N., Genogroup II Noroviruses Efficiently Bind to Heparan Sulfate Proteoglycan Associated with the Cellular Membrane. *J Virol* **2004,** 78, (8), 3817-3826.10.1128/jvi.78.8.3817-3826.2004
172. Rydell, G. E.; Nilsson, J.; Rodriguez-Diaz, J.; Ruvoen-Clouet, N.; Svensson, L.; Le Pendu, J.; Larson, G., Human noroviruses recognize sialyl Lewis x neoglycoprotein. *Glycobiology* **2009,** 19, (3), 309-320
173. Han, L.; Tan, M.; Xia, M.; Kitova, E. N.; Jiang, X.; Klassen, J. S., Gangliosides are ligands for human noroviruses. *J Am Chem Soc* **2014,** 136, (36), 12631-12637.10.1021/ja505272n
174. Almand, E. A.; Moore, M. D.; Jaykus, L. A., Norovirus Binding to Ligands Beyond Histo-Blood Group Antigens. *Frontiers in Microbiology* **2017,** 8,
175. Olival, K. J.; Hosseini, P. R.; Zambrana-Torrelio, C.; Ross, N.; Bogich, T. L.; Daszak, P., Host and viral traits predict zoonotic spillover from mammals. *Nature* **2017,** 546, (7660), 646-650.10.1038/nature22975
176. Caddy, S.; Breiman, A.; le Pendu, J.; Goodfellow, I., Genogroup IV and VI canine noroviruses interact with histo-blood group antigens. *J Virol* **2014,** 88, (18), 10377-10391.10.1128/jvi.01008-14
177. Zakhour, M.; Ruvoën-Clouet, N.; Charpilienne, A.; Langpap, B.; Poncet, D.; Peters, T.; Bovin, N.; Le Pendu, J., The αGal epitope of the histo-blood group antigen family is a ligand for bovine norovirus Newbury2 expected to prevent cross-species transmission. *PLoS Pathog* **2009,** 5, (7).10.1371/journal.ppat.1000504
178. Taube, S.; Perry, J. W.; McGreevy, E.; Yetming, K.; Perkins, C.; Henderson, K.; Wobus, C. E., Murine Noroviruses Bind Glycolipid and Glycoprotein Attachment Receptors in a Strain-Dependent Manner. *J Virol* **2012,** 86, (10), 5584–5593
179. Orchard, R. C.; Wilen, C. B.; Doench, J. G.; Baldridge, M. T.; McCune, B. T.; Lee, Y. C. J.; Lee, S.; Pruett-Miller, S. M.; Nelson, C. A.; Fremont, D. H.; Virgin, H. W., Discovery of a proteinaceous cellular receptor for a norovirus. *Science* **2016,** 353, (6302), 933-936.10.1126/science.aaf1220
180. Yamamoto, F.; Cid, E.; Yamamoto, M.; Saitou, N.; Bertranpetit, J.; Blancher, A., An integrative evolution theory of histo-blood group ABO and related genes. *Sci Rep* **2014,** 4, (6601).10.1038/srep06601.
181. Jones, K. E.; Daszak, P., Global trends in emerging infectious diseases. *Nature* **2008,** 451, 990-994.10.1038/nature06536.

182. Jothikumar, N.; Lowther, J. A.; Henshilwood, K.; Lees, D. N.; Hill, V. R.; Vinjé, J., Rapid and sensitive detection of noroviruses by using TaqMan-based one-step reverse transcription-PCR assays and application to naturally contaminated shellfish samples. *Appl Environ Microbiol* **2005,** 71, (4), 1870-1875.10.1128/aem.71.4.1870-1875.2005
183. Parra, G. I.; Bok, K.; Taylor, R.; Haynes, J. R.; Sosnovtsev, S. V.; Richardson, C.; Green, K. Y., Immunogenicity and specificity of norovirus Consensus GII.4 virus-like particles in monovalent and bivalent vaccine formulations. *Vaccine* **2012,** 30, 3580– 3586
184. Mukti K. Nayak a, G. B. a., Ganesh C. Sahoo a, Rittwika Bhattacharya a, Jan Vinje b, Nobumichi Kobayashi c, Mamta Chawla Sarkar a, Mihir K. Bhattacharya a, Triveni Krishnan, Detection of a novel intergenogroup recombinant Norovirus from Kolkata, India. *Virology* **2008,** 377, (1), 117-123.10.1016/j.virol.2008.04.027
185. Tung, G. P.; Kaneshi, K.; Ueda, Y.; Nakaya, S.; Nishimura, S.; Yamamoto, A.; Sugita, K.; Takanashi, S.; Okitsu, S.; Ushijima, H., Genetic heterogeneity, evolution, and recombination in noroviruses. *J Med Virol* **2007,** 79, (9), 1388-1400.10.1002/jmv.20924
186. Le Guyader, F. S.; Loisy, F.; Atmar, R. L.; Hutson, A. M.; Estes, M. K.; Ruvoën-Clouet, N.; Pommepuy, M.; Le Pendu, J., Norwalk virus-specific binding to oyster digestive tissues. *Emerg Infect Dis* **2006,** 12, (6), 931-936PMC2596755
187. Le Guyader, F. S.; Bon, F.; DeMedici, D.; Parnaudeau, S.; Bertone, A.; Crudeli, S.; Doyle, A.; Zidane, M.; Suffredini, E.; Kohli, E.; Maddalo, F.; Monini, M.; Gallay, A.; Pommepuy, M.; Pothier, P.; Ruggeri, F. M., Detection of multiple noroviruses associated with an international gastroenteritis outbreak linked to oyster consumption. *J Clin Microbiol* **2006,** 44, (11), 3878-3882.10.1128/jcm.01327-06
188. Le Guyader, F. S.; Atmar, R. L.; Le Pendu, J., Transmission of viruses through shellfish: When specific ligands come into play. *Curr Opin Virol* **2012,** 2, (1), 103-110.10.1016/j.coviro.2011.10.029
189. Costantini, V.; Loisy, F.; Joens, L.; Le Guyader, F. S.; Saif, L. J., Human and animal enteric caliciviruses in oysters from different coastal regions of the United States. *Appl Environ Microbiol* **2006,** 72, (3), 1800-1809.10.1128/aem.72.3.1800-1809.2006
190. Becker-Dreps, S.; Cuthbertson, C. C.; Bucardo, F.; Vinje, J.; Paniagua, M.; Giebultowicz, S.; Espinoza, F.; Emch, M., Environmental factors associated with childhood norovirus diarrhoea in León, Nicaragua. *Epidemiol Infect* **2017,** 145, (8), 1597-1605.10.1017/s0950268817000322
191. Wang, Q. H.; Souza, M.; Funk, J. A.; Zhang, W.; Saif, L. J., Prevalence of noroviruses and sapoviruses in swine of various ages determined by reverse transcription-PCR and microwell hybridization assays. *J Clin Microbiol* **2006,** 44, (6), 2057-2062.10.1128/jcm.02634-05
192. Martella, V.; Lorusso, E.; Decaro, N.; Elia, G.; Radogna, A.; D'Abramo, M.; Desario, C.; Cavalli, A.; Corrente, M.; Camero, M.; Germinario, C. A.; Bányai, K.; Di Martino, B.; Marsilio, F.; Carmichael, L. E.; Buonavoglia, C., Detection and molecular characterization of a canine norovirus. *Emerg Infect Dis* **2008,** 14, (8), 1306-1308.10.3201/eid1408.080062
193. Mauroy, A.; Scipioni, A.; Mathijs, E.; Ziant, D.; Daube, G.; Thiry, E., Genetic and evolutionary perspectives on genogroup III, genotype 2 bovine noroviruses. *Arch Virol* **2014,** 159, (1), 39-49.10.1007/s00705-013-1791-7
194. Tse, H.; Lau, S. K. P.; Chan, W. M.; Choi, G. K. Y.; Woo, P. C. Y.; Yuen, K. Y., Complete genome sequences of novel canine noroviruses in Hong Kong. *J Virol* **2012,** 86, (17), 9531-9532.10.1128/jvi.01312-12
195. Tse, H.; Chan, W. M.; Lam, C. S. F.; Lau, S. K. P.; Woo, P. C. Y.; Yuen, K. Y., Complete genome sequences of novel rat noroviruses in Hong Kong. *J Virol* **2012,** 86, (22), 12435-12436.10.1128/jvi.01976-12
196. Symes, S. J.; Allen, J. L.; Mansell, P. D.; Woodward, K. L.; Bailey, K. E.; Gilkersona, J. R.; Browning, G. F., First detection of bovine noroviruses and detection of bovine coronavirus in Australian dairy cattle. *Aust Vet J* **2018,** 96, (6), 203-208.10.1111/avj.12695.
197. Souza, F.; Alfieri, A. A.; Alfieri, A. F.; Lorenzetti, E.; Headley, S. A.; Passos, F.; Silvestre, T.; Zago, L.; Mottin, V.; Montiani-Ferreira, F.; Lange, R.; Svoboda, W.; Gomes, E., The evaluation of enteric viruses in asymptomatic free-ranging non-human primates (Alouatta guariba clamitans, Alouatta caraya, Callithrix spp., Callithrix penicillata, and Leontopithecus caissara) in southern Brazil. *J Med Primatol* **2012,** 41, (5), 304-308.10.1111/j.1600-0684.2012.00557.x
198. Oliver, S. L.; Dastjerdi, A. M.; Wong, S.; El-Attar, L.; Gallimore, C.; Brown, D. W. G.; Green, J.; Bridger, J. C., Molecular characterization of bovine enteric caliciviruses: A distinct third genogroup of noroviruses (Norwalk-like viruses) unlikely to be of risk to humans. *J Virol* **2003,** 77, (4), 2789-2798.10.1128/jvi.77.4.2789-2798.2003

199. Tian, P.; Jiang, X.; Zhong, W.; Jensen, H. M.; Brandl, M.; Bates, A. H.; Engelbrektson, A. L.; Mandrell, R., Binding of recombinant norovirus like particle to histo-blood group antigen on cells in the lumen of pig duodenum. *Res Vet Sci* **2007,** 83, (3), 410-418.10.1016/j.rvsc.2007.01.017
200. Tian, P.; Brandi, M.; Mandrell, R., Porcine gastric mucin binds to recombinant norovirus particles and competitively inhibits their binding to histo-blood group antigens and Caco-2 cells. *Lett Appl Microbiol* **2005,** 41, (4), 315-320.10.1111/j.1472-765X.2005.01775.x
201. Mauroy, A.; Gillet, L.; Mathijs, E.; Vanderplasschen, A.; Thiry, E., Alternative attachment factors and internalization pathways for GIII.2 bovine noroviruses. *J Gen Virol* **2011,** 92, (6), 1398-1409.10.1099/vir.0.030072-0
202. Hutson, A. M.; Atmar, R. L.; Marcus, D. M.; Estes, M. K., Norwalk virus-like particle hemagglutination by binding to H histo-blood group antigens. *J Virol* **2003,** 77, (1), 405-415.10.1128/jvi.77.1.405-415.2003
203. Singh, B. K.; Glatt, S.; Ferrer, J. L.; A, D. K.; Leuthold, M. M.; Dunder, J.; Hansman, G. S., Structural analysis of a feline norovirus protruding domain. *Virology* **2015,** 474, 181-185.10.1016/j.virol.2014.10.028
